# Total Chemical Synthesis of a Functionalized GFP Nanobody

**DOI:** 10.1002/cbic.202200304

**Published:** 2022-08-23

**Authors:** Yara Huppelschoten, Angela F. Elhebieshy, Dharjath S. Hameed, Aysegul Sapmaz, Jens Buchardt, Thomas E. Nielsen, Huib Ovaa, Gerbrand J. van der Heden van Noort

**Affiliations:** ^1^ Oncode Institute and Dept. Cell and Chemical Biology Leiden University Medical Centre Einthovenweg 2 2333 ZC Leiden The Netherlands; ^2^ Global Research Technologies, Novo Nordisk Novo Nordisk Park 2760 Måløv Denmark

**Keywords:** chemical protein synthesis, click chemistry, nanobodies, protein modifications, solid phase peptide synthesis

## Abstract

Chemical protein synthesis has proven to be a powerful tool to access homogenously modified proteins. The chemical synthesis of nanobodies (Nb) would create possibilities to design tailored Nbs with a range of chemical modifications such as tags, linkers, reporter groups, and subsequently, Nb‐drug conjugates. Herein, we describe the total chemical synthesis of a 123 amino‐acid Nb against GFP. A native chemical ligation‐ desulfurization strategy was successfully applied for the synthesis of this GFP Nb, modified with a propargyl (PA) moiety for on‐demand functionalization. Biophysical characterization indicated that the synthetic GFP Nb‐PA was correctly folded after internal disulfide bond formation. The synthetic Nb‐PA was functionalized with a biotin or a sulfo‐cyanine5 dye by copper(I)‐catalyzed azide‐alkyne cycloaddition (CuAAC), resulting in two distinct probes used for functional in vitro validation in pull‐down and confocal microscopy settings.

## Graphical abstract

**The chemical synthesis** of a full‐length nanobody against GFP ready for selective on‐demand functionalization is described. The folded synthetic nanobody is obtained via a native chemical ligation‐desulfurization strategy and converted into probes by copper(I)‐catalyzed azide‐alkyne cycloaddition. The probes bind their target with high affinity which was validated in pulldown‐ and confocal microscopy experiments.

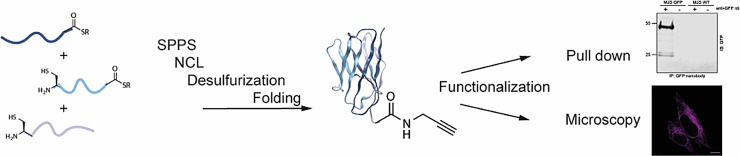

## Introduction

**Figure 1:**
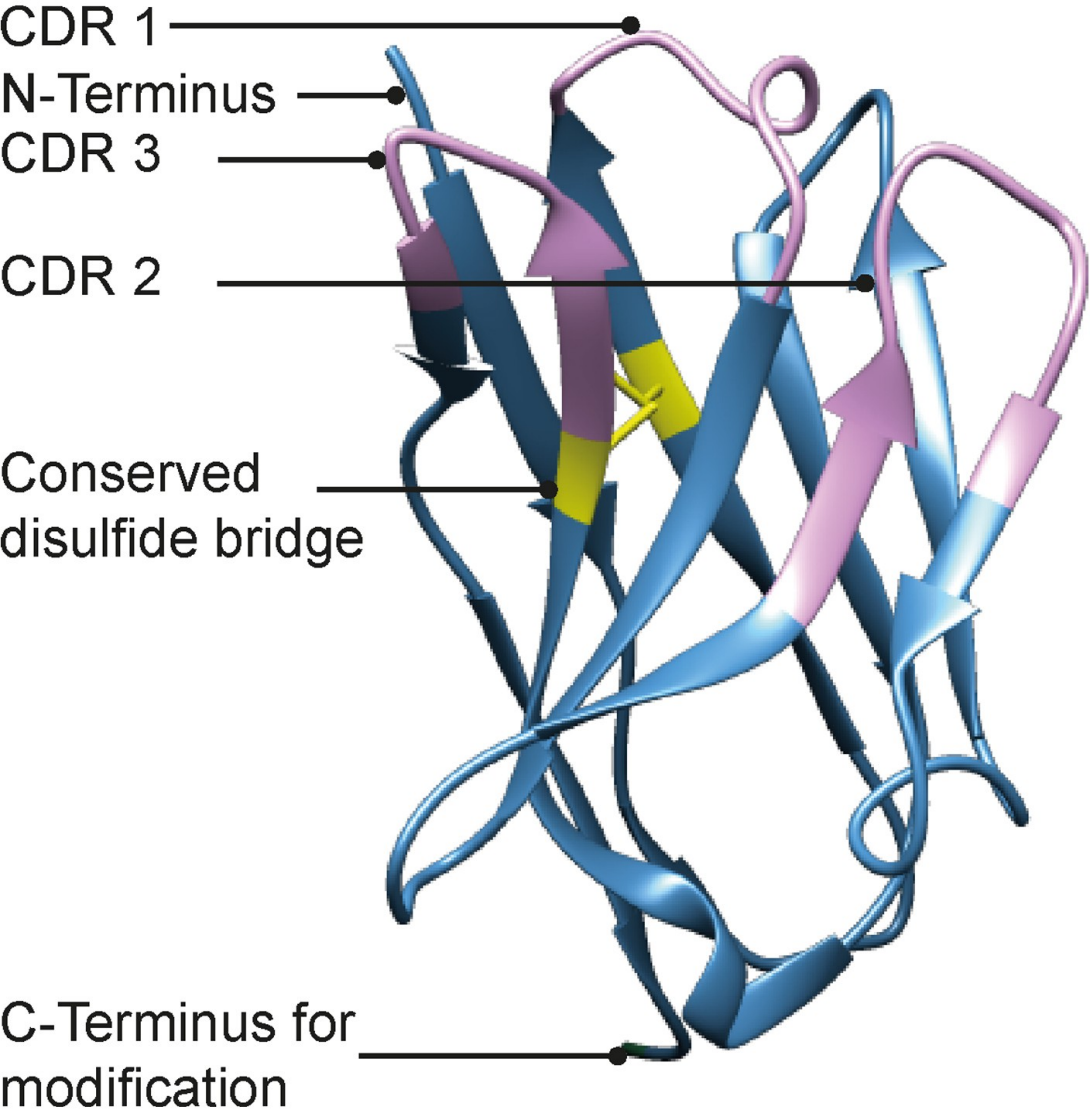
Structure of GFP Nb (PDB: 3OGO). Indicated are the CDR domains in purple, the conserved disulfide bridge in yellow and the C‐terminus as point of modification.

Camelid species produce unique heavy‐chain IgG antibodies existing of a single antigen binding variable heavy‐chain domain (V_H_H) only, also referred to as nanobodies (Nbs).[^1^, ^2^] These Nbs have unique properties such as their small size (∼15 kDa), robustness, high solubility and monomeric nature, all properties that have inspired many researchers to explore them as exquisite research tools in structural‐, cell‐ and developmental biology ever since their serendipitous discovery.[^3^, ^4^, ^5^, ^6^, ^7^, ^8^] In addition, Nbs are seen as promising new therapeutics due to their high affinity (nM range) for their targets, easy tissue penetration, and low immunogenicity.[^9^, ^10^] Accordingly, a lot of interest has been raised for the functionalization of Nbs for various applications such as diagnostic tools, Nb‐drug conjugates, and bivalent Nb conjugates.^[11]^ Conventionally, Nbs are produced via protein expression which provides a functional Nb with ease, albeit with limited modification possibilities such as epitope tagging and other naturally existing mutations. Chemical synthesis of proteins, however, expands the degree of freedom of modification with both natural and unnatural amino acids.^[12]^ The accessibility of synthetic proteins has increased thanks to modern native chemical ligation (NCL) and desulfurization methods.[^12^, ^13^, ^14^] The ability of a Nb to retain target affinity may be drastically compromised as a result of unselective chemical labeling, as is often observed with conventional NHS‐ or maleimide modification reagents. We imagined that, the chemical synthesis of a Nb would accelerate the process of generating homogeneous Nb‐conjugates. Many functional groups suitable for chemoselective labeling would be easily introduced at defined, non‐interfering regions of the Nb through a synthetic approach. The structure of Nbs is highly conserved, making it an attractive protein for generic chemical synthesis, thus paving the way for a modular synthetic approach that, with minor customization, could be broadly applied for a multitude of nanobodies. The general Nb structure comprises nine β‐strands organized in a four‐ and a five‐stranded β‐sheet forming the conserved framework regions (FRs), connected via the complementarity determining region (CDR) loops and a conserved disulfide bond.[^15^, ^16^] The specificity for its target is obtained through the three CDRs at the ends of the variable domains. The long CDR3 loop provides the most significant contribution to the specificity and affinity of the Nb. As a proof‐of‐concept, we selected a nanobody against GFP (referred to hereafter as GFP Nb) aiming to validate a synthetic approach that could prove useful within multiple applications (Figure 1).^[17]^

Considering the importance of the N‐terminal clustering of the CDR loops in the affinity of Nbs towards their targets, we envisioned that incorporating a propargyl moiety at the C‐terminus would be ideal for later modifications using copper mediated azide alkyne cycloaddition (CuAAC) chemistry which uses mild, near physiological reaction conditions.^[18]^ We here present a native chemical ligation‐based synthesis for the generation of a functionalizable GFP Nb. On‐demand conjugation of the synthetic Nb to either an affinity tag or a fluorescent moiety results in easy access to Nb‐conjugates in a divergent manner which can be used as tools in e. g. pull‐down experiments and confocal microscopy.

## Results and Discussion

**Figure 2:**
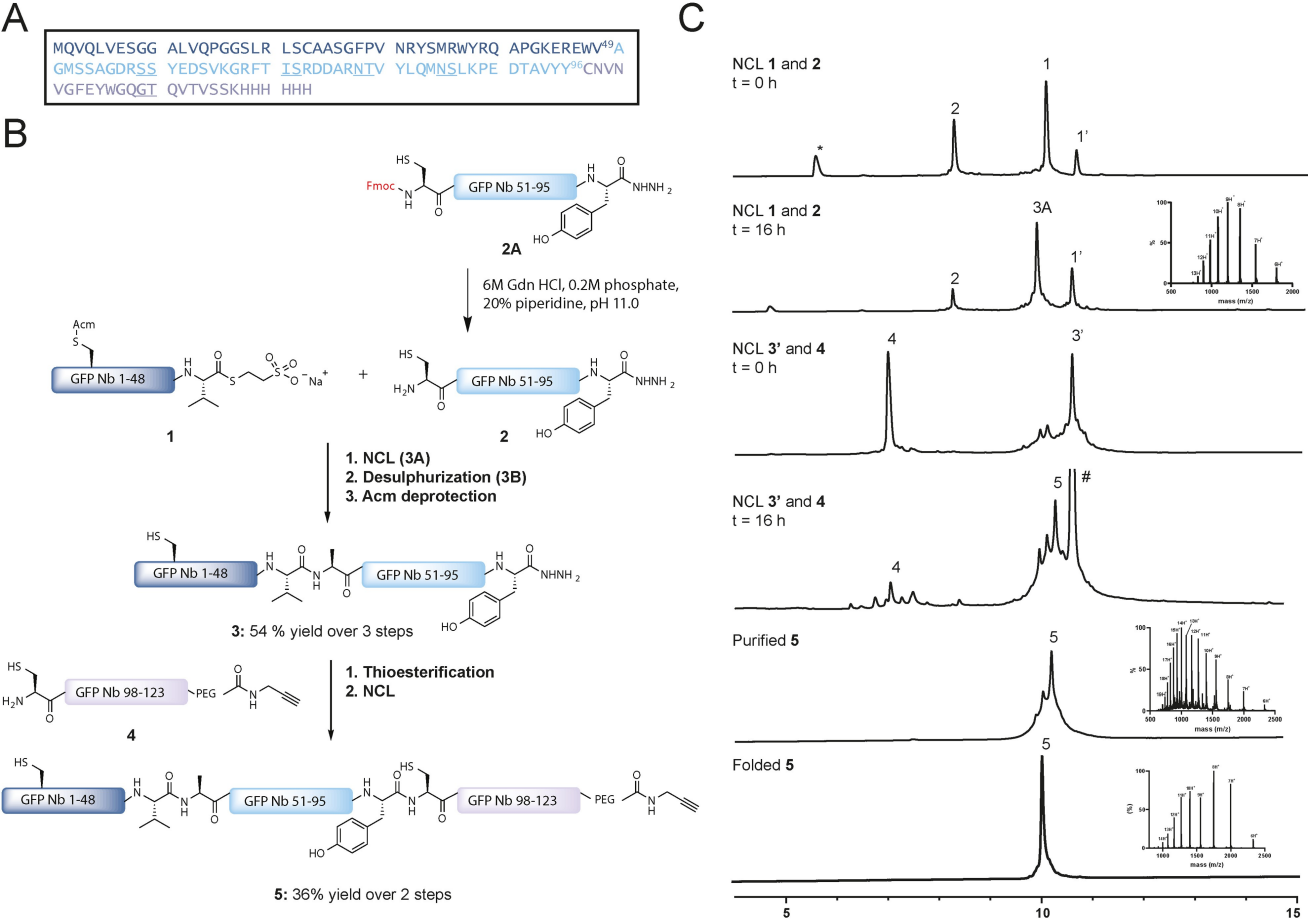
A. Sequence of GFP Nb, with underlined pseudo‐proline dipeptides and iso‐acyl dipeptides used in SPPS. B. Synthetic strategy for obtaining GFP Nb. C. UPLC analysis of the NCL of **1** and **2**, the NCL of **3** and **4**, purification of **5** and folding of **5**. ‘ indicates MPAA thioesters, * indicates dibenzofulvene adduct, # indicates MPAA disulfide adduct.

Although the GFP Nb is relatively small, containing 123 amino acids, the β‐sheet rich structure is known to increase the likelihood of aggregation, on‐resin and in‐solution, during Fmoc‐based solid‐phase peptide synthesis (SPPS).^[19]^ Initial investigations proved that a three‐segment NCL approach was necessary for the synthesis of the GFP Nb.^[20]^ The GFP Nb contains two native Cys residues, of which only one, however, is located at an appropriate potential ligation position (Cys_97_) (Figure 2A). Therefore, NCL‐desulfurization chemistry was chosen to assemble the Nb, as an Ala‐to‐Cys mutation could facilitate a NCL position that could be converted back to the native Ala using radical desulfurization post NCL. Accordingly, we envisioned the use of the acetamidomethyl (Acm) group to protect the other native Cys and prevent unwanted thioesterification or desulfurization during the construction of the GFP Nb. Our strategy for the synthesis of GFP Nb is outlined in Figure 2, where we divided the polypeptide sequence into three fragments. Thioester fragment GFP Nb_1–49_ (**1**), hydrazide fragment Cys‐GFP Nb_50–96_ (**2**), and Cys‐GFP Nb_97–123_ (**4**) were all prepared according to Fmoc‐SPPS strategy on hydrazide or 2‐chlorotrityl resins.^[21]^ Peptide **1** and **2A** were both prepared as hydrazides for subsequent (*in‐situ*) activation and thiolysis. Peptide **1** was synthesized uneventfully with a final yield of 9 % (Figure S1). During the initial attempt, the synthesis and purification of peptide **2** proved to be challenging. In order to increase yield and crude purity, four pseudo‐proline building blocks were incorporated during SPPS (underscored in Figure 2A and Table S2). In addition, to overcome purification difficulties, the Fmoc was retained at the N‐terminus of the peptide, increasing the retention time of the product significantly, compared to the capped deletion sequences formed during synthesis. This resulted in a significant increase in yield (3.6 % yield in the initial attempt for **2** towards 13.2 % yield for **2A**) and enhanced purification efficiency (Figure S2). Furthermore, we experienced a difficult purification of peptide **4** using reversed phase‐HPLC due to low solubility of the peptide most likely caused by the hydrophobic stretch at the N‐terminal end of the sequence. The addition of a GT iso‐acyl dipeptide to **4** (underscored in Figure 2A) increased the solubility and improved the purification process, resulting in pure peptide (7 % yield) (Figure S3).

In 2020, Kar et al.^[22]^ described a one‐pot Fmoc deprotection and NCL strategy which provided an excellent strategy for the first NCL of peptide **1** with peptide **2A**, via *in‐situ* preparation of **2**. Peptide **2A** was first subjected to piperidine treatment at pH 10.7 to furnish the deprotected peptide **2** within 10 minutes (Figure S4). After pH adjustment to pH 7.0, peptide thioester **1** was added to the reaction mixture containing **2** to afford ligated product **3 A** after 16 hours at 37 °C (Figure 2C). Due to the low reactivity of the Val thioester, 4‐mercaptophenylacetic acid (MPAA)^[23]^ was used as additive in this ligation and afterwards removed using a 3 kDa molecular weight cutoff spin filter, monitoring the MPAA removal by LC–MS/UV chromatography (Figure S5). Subsequently, the Cys residue was desulfurized by applying radical desulfurization conditions (TCEP, VA‐044 and GSH) leading to **3B** after 16 hours (Figure S6).^[14]^

The integrity of the propargyl moiety on peptide **4** is not compatible with Pd chemistry, therefore we opted to conduct the Acm removal in **3B** before the final ligation step between peptide **3B** and **4**. We envisioned that the use of PdCl_2_ for the Acm removal of protected Cys_23_ in **3B** would be a good option due to its compatibility with the denaturing 6 M Gdn buffer.^[24]^ All chemicals from the desulfurization reaction were removed using a 3 kDa cutoff spin filter before the addition of PdCl_2,_ and the reaction was completed within 1 hour and subsequently quenched with DTT, prior to purification of the peptide using size‐exclusion chromatography (SEC), to obtain product **3** in a 54 % (Figure S5). We used SEC as an alternative purification method using the high chaotropic NCL buffer, 6 M Gdn buffer, as the purification of fragment **3** proved to be troublesome using standard reversed‐phase HPLC due to its hydrophobic nature. Before we continued with the second ligation, peptide **4A** was incubated at pH 7–8 for 10 minutes to obtain the native peptide bond after an O→N acyl shift (Figure S7). Followed by the one‐pot thioesterification of **3** and ligation to **4**, final product **5** was obtained in 36 % yield (Figure 2C, Figure S8–9).

**Figure 3:**
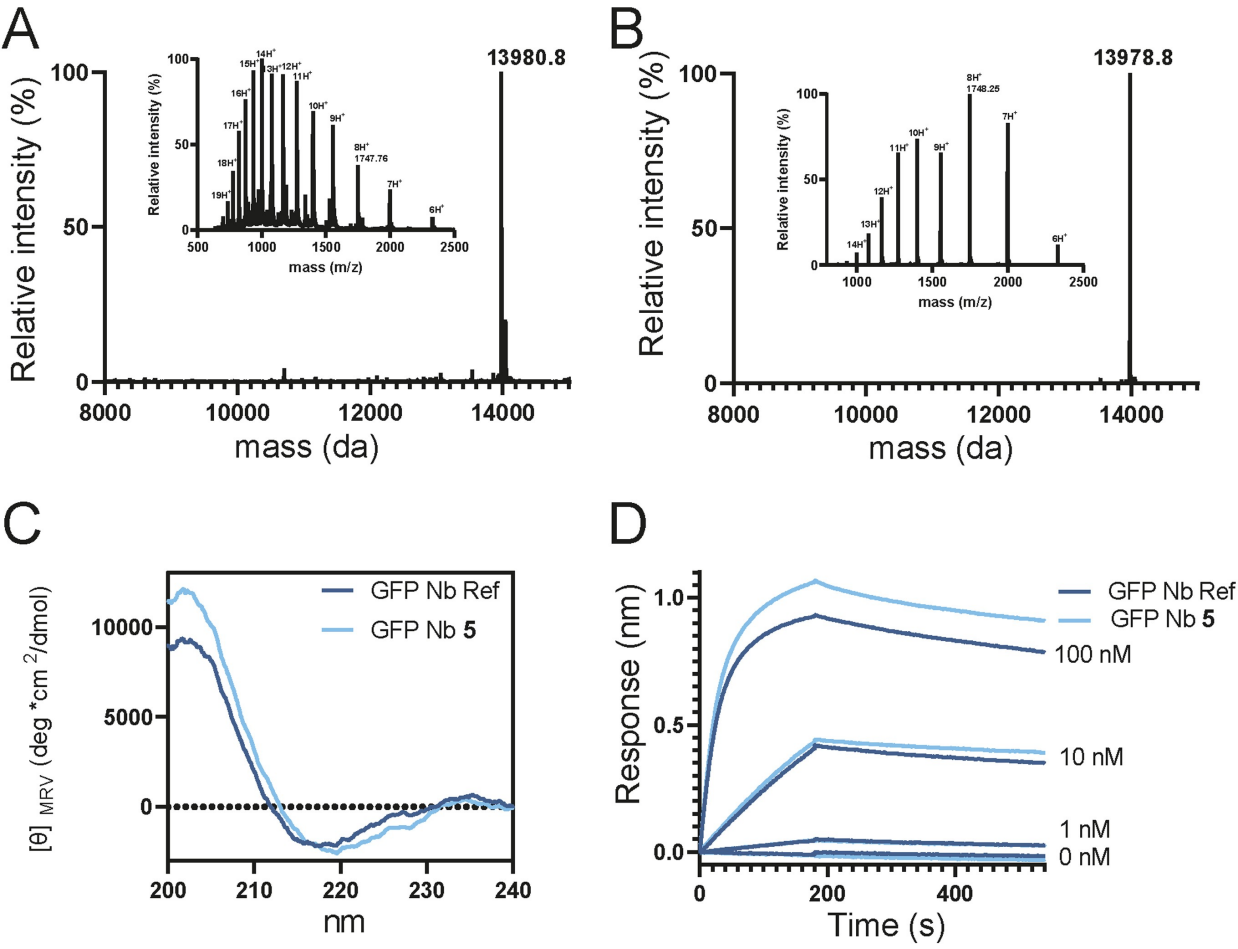
A. Deconvoluted mass spectrum and ESI Mass spectrum (inset) of unfolded **5**. B. Deconvoluted mass spectrum and ESI Mass spectrum (inset) of folded **5**. C. Circular dichroism spectra comparing recombinant and synthetic GFP Nb. D. Bio Layer Interferometry analysis of folded **5** in comparison to expressed GFP Nb.

With the full‐length GFP Nb_1–123_‐linker‐PA in hand, we continued to the folding step including disulfide bond formation. The folding was carried out by stepwise dialysis in phosphate buffered saline (PBS). As spontaneous disulfide formation did not occur within 48 hours in PBS we added 2,2’‐Dithio‐dipyridin (DTP),[^25^, ^26^] a known disulfide bond formation accelerator. Indeed, after the addition of 1 mM DTP to the folding buffer, initiation of disulfide bond formation was observed after 1 hour and completed within 16 hours as was clearly observed in the high‐resolution mass spectrum by the loss of 2 Da (Figure 3B).

To confirm the proper folding of the chemically synthesized and folded GFP Nb (**5**), we conducted circular dichroism (CD) experiments. The synthetic GFP Nb exhibited absorptions of β‐sheet structures similar to the recombinantly expressed GFP Nb,^[17]^ indicating that their folding is comparable (Figure 3C). In addition, biolayer interferometry (BLI) experiments were performed to determine the affinity of the synthetic GFP Nb **5** to its target protein GFP and compared to that of the recombinantly expressed GFP Nb. The C‐terminal His‐tag on both the expressed and synthetic Nb was used to immobilize the Nbs on Ni‐NTA biosensor tips and untagged GFP was used as the analyte at concentrations ranging from 1 nM to 100 nM. With this setup, the expressed and synthetic GFP Nb showed similar binding profiles (Figure 3D) and affinities of 1.12 and 1.11 nM, respectively. These values are in agreement with previously reported data in literature (1.4 nM).^[17]^

**Figure 4:**
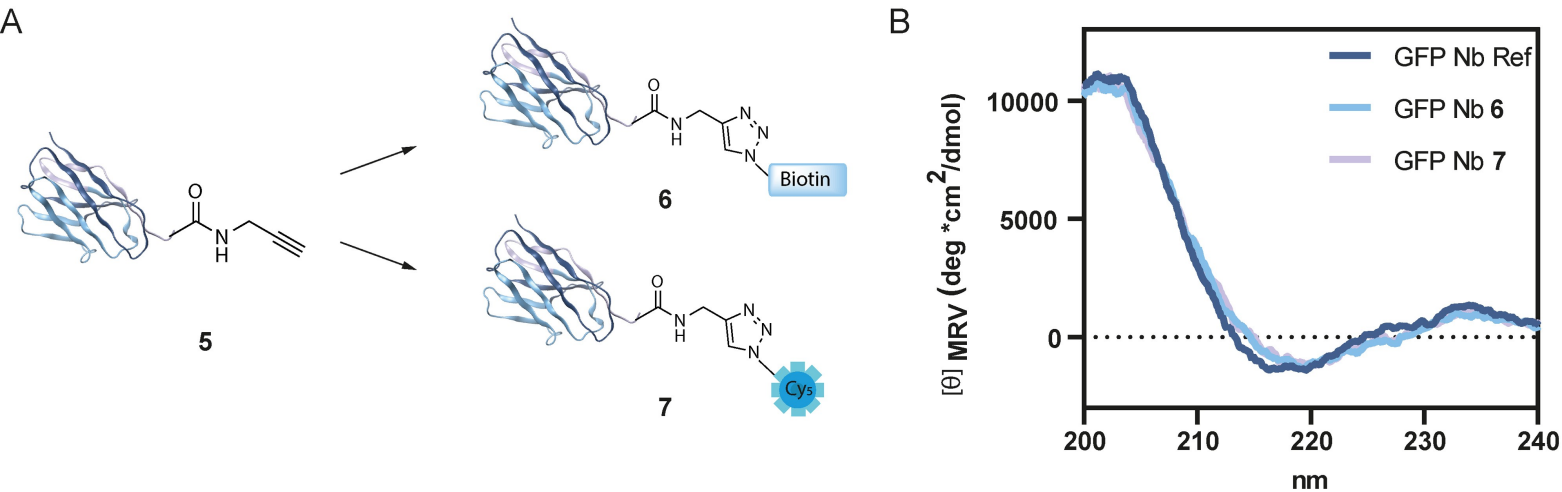
A. Functionalization of synthetic Nb **5**. Nanobody structure PDB ID: 3OGO.^[17]^ B. circular dichroism of expressed GFP Nb, synthetic GFP Nb conjugates **6** and **7**.

Next, we wanted to show the adaptability of the fully synthetic Nb as a chemical tool by modifying the C‐terminal propargyl moiety using a bio‐orthogonal labeling strategy. Accordingly, we used copper(I)‐catalyzed alkyne‐azide cycloaddition (CuAAC) chemistry to functionalize the synthetic Nb with an azide‐functionalized biotin molecule for the purpose of pull‐down experiments (Figure 4A). Unfolded, purified **5** was reacted with biotin‐azide using mild reaction conditions (3 mM CuSO_4_, 10 mM sodium ascorbate, and 2 mM tris‐(hydroxypropyltriazolylmethyl)amine (THPTA)) to form the GFP Nb‐biotin conjugate (**6**) (Figure S11). After the CuAAC, the synthetic Nb was folded as described previously for compound **5**, removing all additives from the CuAAC reaction during the dialysis step. In addition, we envisioned that modification of **5** with sulfo‐Cyanine5‐azide (Cy5) would lead to the opportunity to validate the proper functioning and target binding of our Nb by co‐localization of the Nb with GFP tagged proteins in cells using confocal microscopy. Hence, we used the same procedure to synthesize a GFP Nb‐Cy5 conjugate (**7**) (Figure S12). Next, we measured CD to warrant correct folding of the functionalized Nbs (Figure 4B) and continued with CD denaturing experiments to investigate the stability of the synthetic Nb‐conjugates compared to the expressed GFP Nb. Both Nb‐conjugates showed a similar denaturing pattern as the expressed GFP Nb indicating that introduction of C‐terminal cargo onto the Nb did not alter its biophysical properties (Figures S14–S16).

**Figure 5:**
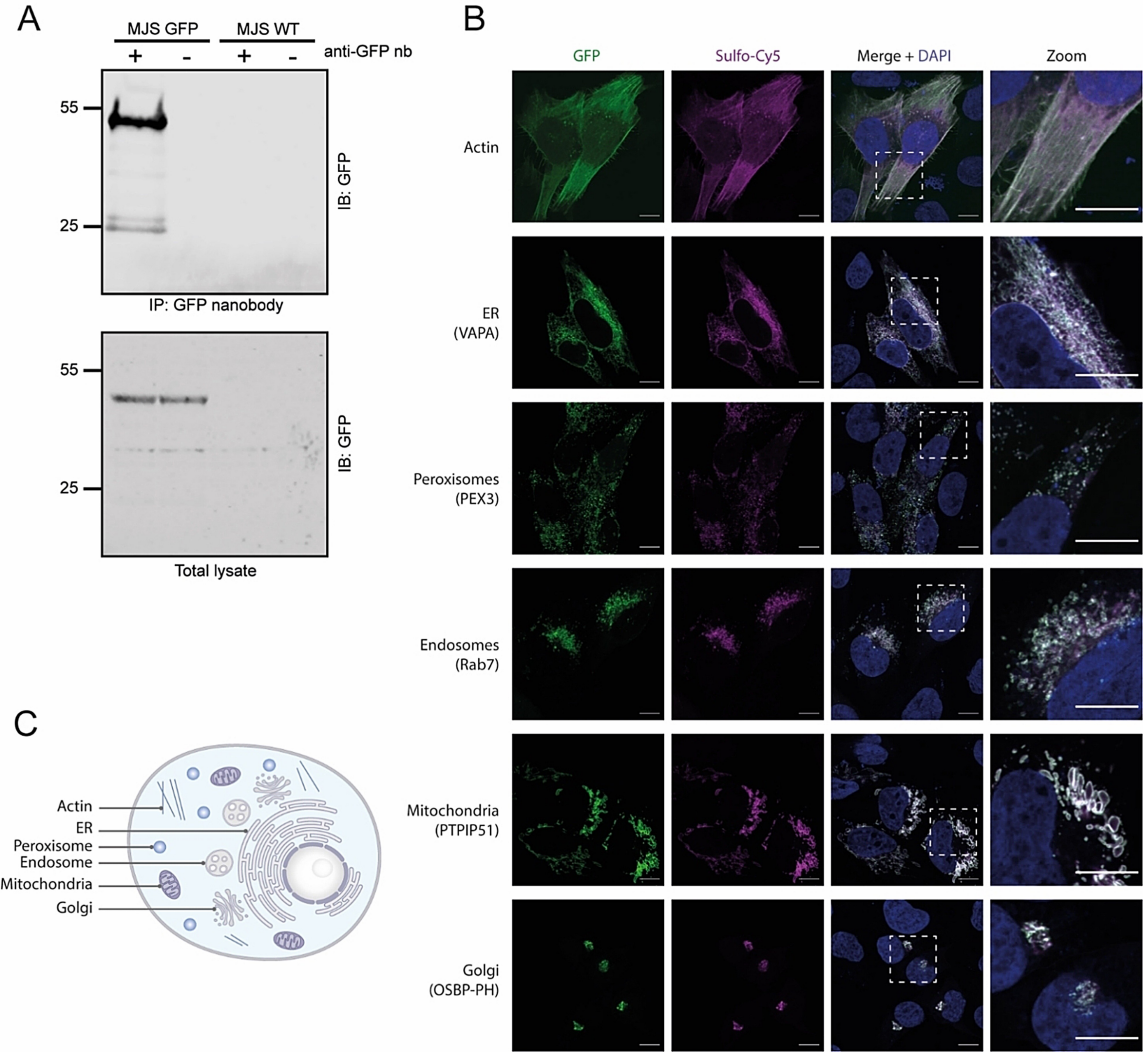
A. Western blot analysis of the pull‐down of GFP‐Rab7 from cell lysate using GFP Nb‐biotin conjugate **6**. The signal around 25 kDa is GFP as a result of protein degradation. B. Confocal images of MelJuSo cells expressing GFP‐Rab7 in the presence of GFP Nb‐Cy5 **7**. C. Illustration of a cell highlighting the cellular compartments visualized in B.

We wanted to test Nb conjugates **6** and **7** in *in vitro* assays for recognition of GFP labeled proteins. First, we set out to validate the binding of the synthetic GFP Nb to GFP‐fusion proteins in a complex protein mixture by performing a pull‐down assay. For this purpose, we decided to use the MelJuSo cell line established in our lab^[27]^ that stably expresses GFP‐tagged small GTPase Rab7 that is a central regulator of membrane trafficking in multiple directions.^[28]^ Hence, we incubated the GFP Nb‐biotin conjugate (**6**) with MelJuSo cell lysate expressing GFP‐Rab7 to perform a pull‐down assay. As negative control to ensure GFP‐specific binding, we used WT MelJuSo cells that did not express GFP‐Rab7. After two hours of incubating conjugate **6** with the cell lysates of both GFP‐Rab7 MelJuSo or WT MelJuSo, we were able to selectively pull‐down the GFP‐Rab7 protein from the cell lysate showing a signal around 55 kDa, equal to the molecular weight of GFP‐Rab7 (Figure 5A). Negligible background signal confirms the selectivity of the synthetic GFP Nb for GFP over other proteins present in the cell lysate (Figure S18).

Next, we continued with confocal microscopy validation, and used GFP Nb‐Cy5 conjugate **7** to examine co‐localization with GFP tagged proteins located in different cellular compartments (cytoskeleton, endoplasmic reticulum (ER), peroxisomes, endosomes, mitochondria and Golgi). MelJuSo cells were hence transfected with six different plasmids encoding for GFP tagged Actin, VAMP‐Associated Protein A (VAPA), Peroxisomal biogenesis factor 3 (PEX3), Rab7, Protein tyrosine phosphatase interacting protein 51 (PTPIP51) and Oxysterol binding protein (OSBP‐PH) proteins (Figure 5B and C). The cells were fixed and the membranes were permeabilized before incubation with the conjugate **7** for 1 hour. The incubation of the transfected MeIJuSo cells with **7** resulted in a complete overlap between the GFP signal and the Cy5 signal, indicating the co‐localization of the synthetic Cy5‐Nb with the GFP tagged proteins (Figure 5B). The staining with conjugate **7** resulted in a strong signal in each of the tested cell compartments with minimal background, indicating full target engagement of our synthetic Nb (Figure 5B). The ability to visualize GFP fusion proteins at various cellular locations further efficiently showcases the broad applicability of the synthetic Nb.

## Conclusion

In conclusion, we have developed a practical native chemical ligation‐based synthetic approach for the generation of a synthetic GFP Nb ready for on‐demand functionalization. With this method, we can obtain homogenous batches of labeled Nb by selectively labeling of the Nb using CuAAC without altering the properties of the Nb, such as folding or thermo‐stability. This technology was successfully applied to modify the GFP‐Nb with both a biotin or a fluorophore which were used in pull‐down and confocal microscopy experiments, respectively. The Nb labeling is performed without compromising the antigen‐binding site, making this method also applicable for Nbs containing a Cys residue in their CDR domains. In addition, easy modification of the CDR3 domain can be obtained because it is introduced in one of the final synthesis steps. We envision that with this protocol in hand, Nbs against other targets might be synthesized, using a similar strategy, due to the high sequence and structure similarities between Nbs. This methodology could potentially also be applied to the streamlined preparation of Nb drug‐conjugates or (heterogeneous) Nb multimers. Moreover, unnatural amino acids can be easily introduced through the SPPS protocol, e. g. to confer stability of the Nb against degradation *in vivo* and improve the applicability of this protein scaffold.[^29^, ^30^]

## Experimental Section

The data that support the findings of this study are available in the Supporting Information of this article.

## Conflict of interest

The authors declare no conflict of interest.

1

## Supporting information

As a service to our authors and readers, this journal provides supporting information supplied by the authors. Such materials are peer reviewed and may be re‐organized for online delivery, but are not copy‐edited or typeset. Technical support issues arising from supporting information (other than missing files) should be addressed to the authors.

Supporting Information

## Data Availability

The data that support the findings of this study are available in the supplementary material of this article.
