# Supplementary material for: Total Chemical Synthesis of a Functionalized GFP Nanobody
Source: Chembiochem. 2022 Aug 23;23(19):e202200304. doi: 10.1002/cbic.202200304 (PMC9804225; doi:10.1002/cbic.202200304)
Supplement: Supplementary file 1 — Supporting Information [file CBIC-23-0-s001.pdf]

ChemBioChem

Supporting Information

Total Chemical Synthesis of a Functionalized GFP Nanobody

Yara Huppelschoten[+], Angela F. Elhebieshy[+], Dharjath S. Hameed, Aysegul Sapmaz, Jens Buchardt, Thomas E. Nielsen, Huib Ovaa, and Gerbrand J. van der Heden van Noort*

Supporting Information

Table of contents

General procedures 3
Materials and solvents 3
Analytical methods 3
Solid Phase Peptide Synthesis (SPPS) 4
Preloading 2-chlorotrityl resin 4
Automated Fmoc SPPS 4
Global deprotection from the resin and side chain deprotection 4
Preparative HPLC purification 4
Nanobody characterization 5
Cell culture and pull-down of overexpressed GFP-Rab7 5
Confocal microscopy 5
Bio Layer Interferometry-measurements 6
Synthesis strategy 7
Sequences 8
Preparation of peptide fragments 8
Assembly of GFP Nb 12
Folding of 5 21
CuAAC chemistry on 5 23
Circular dichroism 26
Bio Layer Interferometry 29
Pull-down 29
References 30

General procedures

Materials and solvents

Reagents were obtained from Sigma-Aldrich of the highest available grade and used without further purification. Standard Fmoc-protected amino acid derivatives were used and purchased from Gyros Protein Technologies unless mentioned otherwise. Fmoc-Cys(Acm)-OH and resins for SPPS were obtained from Novabiochem (Merck Millipore), Apigenex and PCAS Biomatrix. Pseudo-proline dipeptides were obtained from Corden Pharma or Bachem. Iso-acyl dipeptides were obtained from AAPPTec. Solvents for SPPS were obtained from Biosolve. VA-044 was procured from Wako Pure Chemical Corporation. Oxyma Pure® was purchased from Gyros Protein Technologies. HPLC grade acetonitrile was obtained from Merck.

Analytical methods

LC-MS conditions

LC-MS measurements were performed on a Waters Acquity UPLC H Class system, Waters Xevo G2-XS QTof with a Waters Acquity BEH 300 Å, C4, 1.7 µm, 2.1 mm x 50 mm (0.4 mL/min). Samples were run at 60 °C using 3 mobile phases: A = 0.1 % formic acid in MilliQ water, B = 0.1 % formic acid in acetonitrile and C = 0.01 % TFA in MilliQ water with a gradient of 5 to 25% B over 1 min, 25 to 65 % B over 6 min followed by 65 to 95 % B over 0.5 min maintaining a composition of 5% C throughout. Data processing was performed using Waters MassLynx Mass Spectrometry Software V4.2 (deconvolution with MaxEnt I function).

Analytical UPLC conditions

UPLC measurements were performed on a Waters Acquity UPLC H Class system with a Waters Acquity BEH 300 Å, C4, 1.7 µm, 2.1 mm x 100 mm (0.4 mL/min). Samples were run at 40 °C using 2 mobile phases: A = 0.05 % TFA in MilliQ water and B = 0.05 % TFA in acetonitrile with a gradient of 5 to 50 % B over 20 min followed 50 to 95% B over 0.5 min. Data processing was performed using Empower software.

Quantification

Charged Aerosol Detection (CAD)

Purified samples were quantified using a Thermo Scientific Vanquish, Corona Veo CAD. Samples were run Acquity BEH 300 Å, C4, 1.7 µm, 2.1 mm x 50 mm at 40 °C using 2 mobile phases: A = 0.1 % TFA in MilliQ water and B = 0.1 % TFA in acetonitrile with a gradient of 0 to 80 % B over 7 min.

Solid Phase Peptide Synthesis (SPPS)

Preloading 2-chlorotrityl resin

2-Chlorotrityl resin (0.57 mmol/gram) was swollen in dry DCM for 30 minutes. A solution of Fmoc-AA-OH (1 equiv.) in dry DCM and DIPEA (4 equiv.) were added, and the resin was shaken for 30 minutes. The resin was washed with DCM twice before capping the remaining trityl groups with methanol/DIPEA/DCM 17:2:1, v/v/v. The resin was dried *in vacuo* prior to determination of the estimated loading of the first amino acid.

Automated Fmoc SPPS

SPPS was performed on a Symphony X (Gyros Protein Technologies) automated peptide synthesizer using standard 9-fluorenylmethoxycarbonyl (Fmoc) based SPPS. Fmoc deprotection was achieved with 2 x 10 min. treatment of 20 vol. % piperidine, 0.1 % Oxyma Pure® in DMF. Peptide couplings were performed using DIC/Oxyma. Amino acid/Oxyma solutions (0.3 M/0.3 M in DMF) were added to the resin at 4-6-fold excess together with equal equivalents of DIC (1.5 M in DMF). The coupling time was 2 hours unless specified otherwise. All dipeptide building blocks were coupled for 4 hours. The residual free amino groups after the coupling reaction were capped by the addition of collidine (3.3 equiv., 1.5 M in DMF) and acetic anhydride (11 equiv., 1.0 M in DMF) and were reacted for 20 minutes. After the final Fmoc deprotection the resin was washed with DMF and DCM.

Global deprotection from the resin and side chain deprotection

Polypeptide sequences containing a cysteine residue were detached from the resin and deprotected by treatment with Reagent K (TFA/phenol/H_2O/thioanisole/EDT, 82.5:5:5:5:2.5 v/v/v/v/v) for 2-3 hours followed by precipitation in ice cold diethylether and collection by centrifugation. Polypeptide sequences containing methionine residues were detached from the resin and deprotected by treatment with TFA/TIPS/H_2O/DCM/NH_4I/DTT, 87:5:2.5:2.5:0.5:2.5, v/v/v/v/v for 2-3 hours followed by precipitation in ice cold diethylether and collection by centrifugation. The pellet was resuspended in diethylether before being collected by centrifugation again. The pellet was dissolved in and lyophilized from H_2O/CH_3CN/AcOH, 65:25:10, v/v/v before purification.

Preparative HPLC purification

Preparative purification was performed on a Gilson HPLC system using a reversed phase HPLC column as specified in the experimental section. Elution was performed using 2 mobile phases: A = 0.1 % TFA in MilliQ water and B = 0.1% TFA in acetonitrile using a linear gradient. Fractions were collected using a Gilson fraction collector. Relevant fractions were assessed by LC-MS and pure peptide was pooled and lyophilized.

Nanobody characterization

The construct for GFP Nb was obtained from add gene (49172) and expressed as described previously by Kubala et al.[1] In brief, protein expression was conducted in E. coli strain BL21(DE3) in a flask containing LB medium and grown to an OD_{600} of 0.5 at 37°C, and then, protein expression was induced using 0.5 mM IPTG. Further fermentation was carried out at 20°C for 20 h. Resultant cell mass was harvested by centrifugation, disrupted by sonication, and subjected to centrifugation to remove cell debris. The cleared cell lysate was subjected to HisTrap affinity purification followed by size-exclusion fractionation (Superdex 75) using an Akta Purifier FPLC system (GE Healthcare).

Cell culture and pull-down of overexpressed GFP-Rab7

MelJuSo (human melanomas) cell lines stably expressing GFP-Rab7 were kindly gifted by A. Sapmaz (LUMC, Leiden) and WT MelJuSo cells, kindly provided by Prof. G. Riethmuller (LMU, Munich).[2] The cells were lysed in lysis buffer (0.8 % NP40, 150 mM NaCl, 50 mM Tris-HCl pH 8.0, 0.05 mM $MgCl_2$ + protease inhibitor) followed by brief sonication. Cell debris was removed by centrifugation. Next, 5 µg of biotin tagged synthetic GFP Nb was added to cell lysates of both GFP-Rab7 expressing cells and WT cells and incubated by rotating for 2 hours at 4 °C. Thereafter, high capacity neutravidin beads (Thermo Scientific, Cat# 29202) were added and incubated by rotating for 1 hour at 4 °C. The beads were extensively washed with lysis buffer and after completely removing the washing buffer, SDS sample buffer supplemented with 2-mercaptoethanol was added to the beads and boiled at 95ºC. The proteins were separated by SDS-PAGE followed by western blotting and detection by ponceau s followed by antibody staining using rabbit anti-GFP antibody[3] followed by IRDye 800CW goat anti-rabbit IgG (H + L) (Li-COR, Cat# 926-32211). The signal was detected using direct imaging by the Odyssey Classic imager (LI-COR).

Confocal microscopy

MelJuso cells were seeded into 24-well plates containing glass coverslips to achieve 40-50% confluency the following day. Cells were transfected with the DNA plasmids in table 1 using X-tremeGENE HP (Roche Cat# XTGHP-RO) according to manufacturer's instruction and cultured for 18-24 hours. Next, the cells were fixed in 3.7% formaldehyde in PBS for 20 min and subsequently permeabilized using 0.1% Triton X-100 in PBS for 10 min. After permeabilization, cells were blocked using 5% (w/v) skim milk powder in PBS for 30 min and incubated with 1 µg Cy5-labelled synthetic Nb (**7**) in blocking buffer for 1 hour at RT. Next, cells washed and were mounted using ProLong Gold antifade Mounting medium with DAPI (Life Technologies, Cat# P36941). Samples were imaged using Leica SP5 or SP8 microscopes equipped with appropriate solid-state lasers, HCX PL 63 times magnification oil emersion objectives and HyD detectors. Image processing and colocalization analyses were performed using the Fiji software.

Table 1. DNA plasmids used for transfection with the corresponding protein.

Protein	Cell compartment	Plasmid	Reference and cloning
RAB7A	Endosomes	GFP-RAB7A	[4]
VAMP-Associated Protein A (VAPA)	ER	GFP-VAPA	[5]
Peroxisomal biogenesis factor 3 (PEX3)	Peroxisomes	PEX3*-SBP-GFP	PEX3*-SBP-GFP was a gift from Juan Bonifacino (Addgene plasmid # 120174).[6]
Protein tyrosine phosphatase interacting protein 51 (PTPIP51)	Mitochondria	PTPIP51-GFP	PTPIP51 ORF was cloned into GFP-C1 vector from PTPIP51-RFP using HindIII and BamHI restriction enzymes.[5]
Oxysterol binding protein (OSBP-PH)	Golgi	EGFP-OSBP-PH	EGFP-OSBP-PH was a gift from Marci Scidmore (Addgene plasmid # 49571).[7]
LifeAct	Actin filaments	Lifeact-EGFP	Addgene Plasmid # 58470

Bio Layer Interferometry-measurements

BLI measurements were performed on an OctetRed system (ForteBio). 100 nM of the expressed GFP Nb or the synthetic GFP Nb were loaded on Ni-biosensors for 2 minutes and washed in binding buffer (phosphate-buffered saline (PBS), 0.05 % Tween-20, 0.01 % BSA, pH 7.4). Thereafter, the sensors were transferred into solutions containing varying concentrations of GFP (100 – 1 nM) to measure the association of the analyte for 3 minutes. Subsequently, the dissociation of the complex was measured in binding buffer for 6 minutes. Dissociation constants (Kd) were calculated using the ForteBio Data Analysis software by co-fitting all concentrations simultaneously.

Synthesis strategy

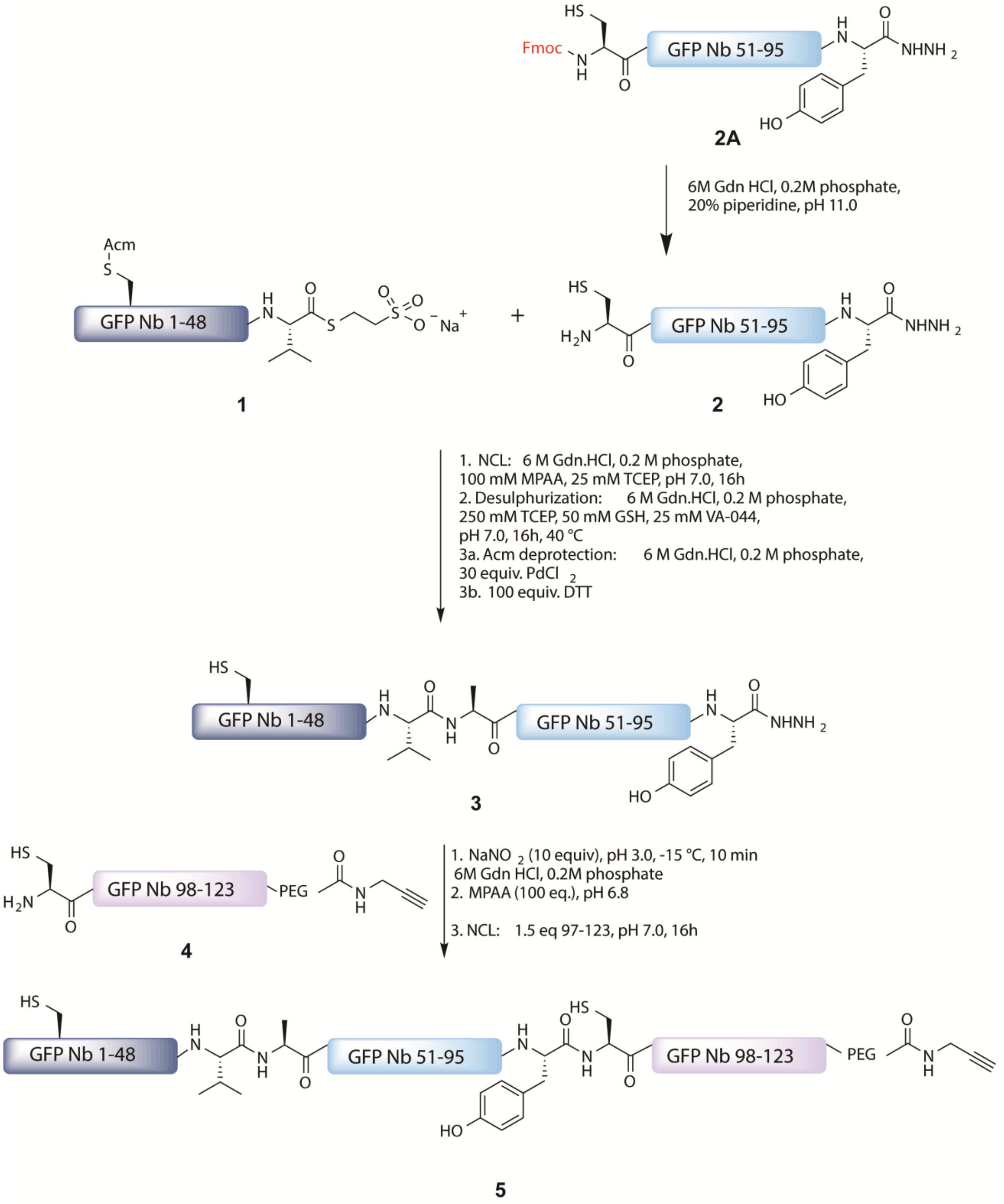

Scheme S1. Complete synthetic approach towards synthetic GFP Nb using NCL-desulfurization chemistry.

Sequences

GFP Nanobody:

1 **MQVQLVESGG ALVQPGGSLR LSCAASGFPV NRYSMRWYRQ**
41 **APGKEREWVA GMSSAGDRSS YEDSVKGRFT ISRDDARNTV**
81 **YLQMNSLKPE DTAVYYCNVN VGFEYWGQGT QVTVSSKHHH HHH**

Table S2. Amino acid sequences of the Nb fragments, psuedoprolines are underscored and iso-acyl dipeptides in bold.

**Segment ID**	**SPPS sequence**
GFP 1-48	H-MQVQLVESGGALVQPGGSLRLSC(Acm)AASGFPVNRYSMRWYRQAPGKEREWV-NHNH$_2$
Fmoc-GFP 49-96	H-CGMSSAGDRSSYEDSVKGRFTISRDDARNTVYLQMNSLKPEDTAVYY-NHNH$_2$
GFP 98-123 OEG	H-CNVNVGFEYWGQ**GT**QVTVSSKHHHHHHX-OH

X was incorporated as a Fmoc-Peg2-OH. Underlined dipeptide sequences were coupled as the respective pseudoproline dipeptides or DMB dipeptides. Italic dipeptides were coupled as the respective iso-acyl dipeptides.

Preparation of peptide fragments

Synthesis of GFP 1-48 thioester 1

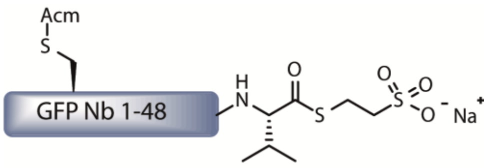

1

The synthesis was performed following general procedures using 2-chlorotrityl hydrazine resin (1.0 gram, 0.32 mmol/gram). The peptide was cleaved from the resin according to the general procedures and purified by preparative HPLC using a Phenomenex, Luna 100 Å, C8(2), 10 µm, 30 mm x 250 mm column (25 to 35%B over 20 min, 30mL/min). Peptide **1** was dissolved in 45 mL of 6 M Gdn.HCl pH 3.0 and 1 M $NaNO_2$ in MilliQ (3.2 mL, 3.2 mmol, 10 equiv.) was added and stirred for 15 min at 0 °C. The reaction was warmed to room temperature and MESNa (5.1 gram, 32 mmol, 100 equiv.) in 6 M Gdn.HCl, 0.2 M phosphate pH 7.0 was added. The pH was adjusted to pH 7.0 and the solution was stirred for 60 min. before purification by preparative RP-HPLC using Phenomenex, Luna 100 Å, C8(2), 10 µm, 30 mm x 250 mm (22 to 32 % B over 45 min, 30mL/min) followed by lyophilization afforded peptide **1** as a white solid (171.6 mg, 9.3 % yield).

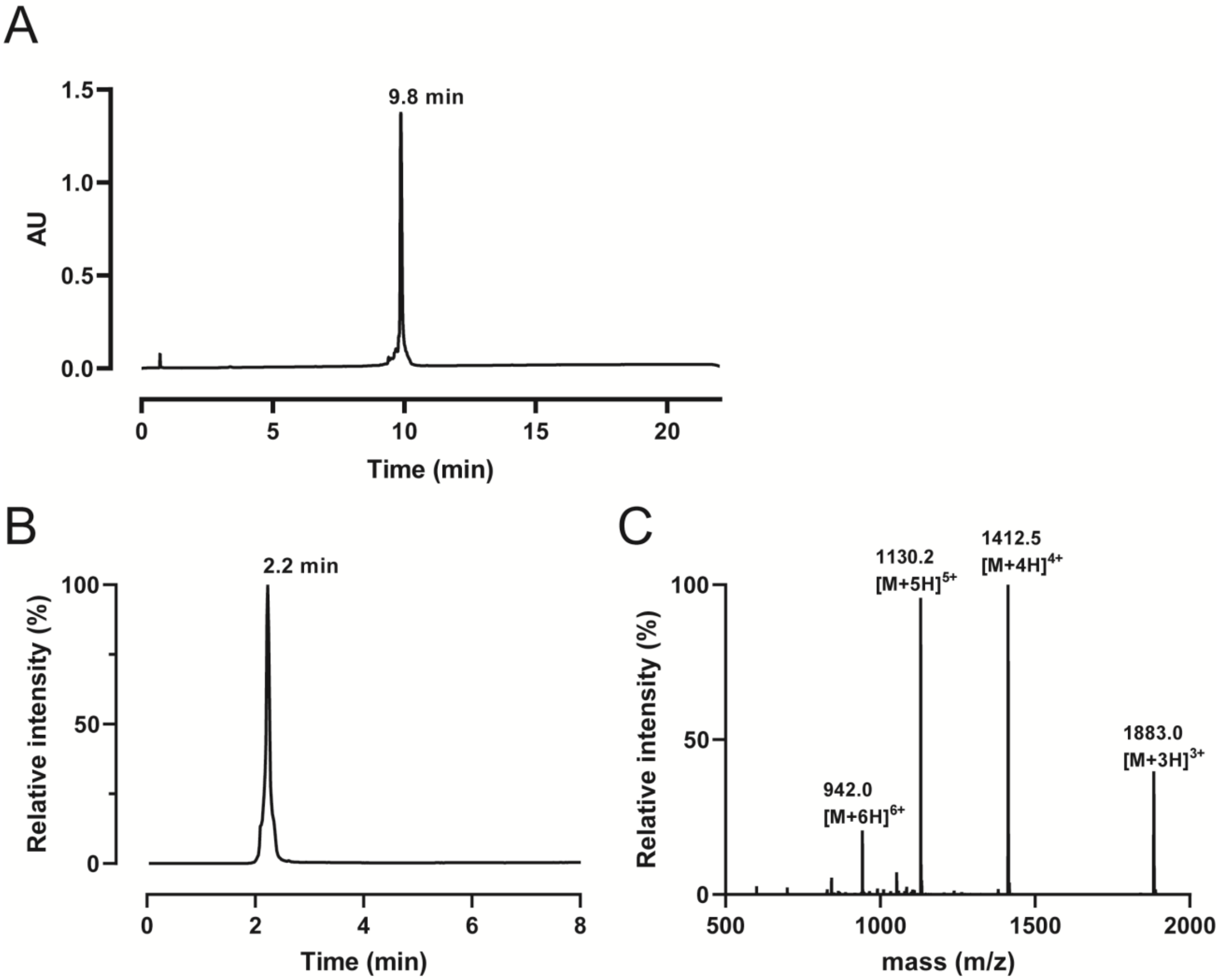

Figure S1. A. UPLC UV chromatogram of purified **1**, Rt 9.86 min. **B.** Total ion chromatogram (LC-MS method C4) of purified **1**, Rt 2.23 min. **C.** Observed ESI spectrum of purified **1**. Calculated Mass (average isotope composition): 5645.74; Observed: $[M + 3H]^{3+}$: 1882.9, $[M + 4H]^{4+}$: 1412.4, $[M + 5H]^{5+:}$ 1130.1, $[M + 6H]^{6+}$: 941.9.

Synthesis of Fmoc-GFP 49-96 hydrazide 2A

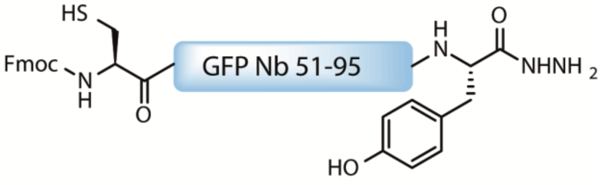

2A

The synthesis was performed following general procedures using 2-chlorotrityl hydrazine resin (1.27 gram, 0.32 mmol/gram). The peptide was cleaved from the resin according to the general procedures and purified by preparative RP-HPLC using a Phenomenex, Luna 100 Å, C8(2), 10 µm, 30 mm x 250 mm column (26 to 33 % B over 40 min, 30 mL/min) followed by lyophilization afforded peptide **2A** as a white solid (296.6 mg, 13.2% yield).

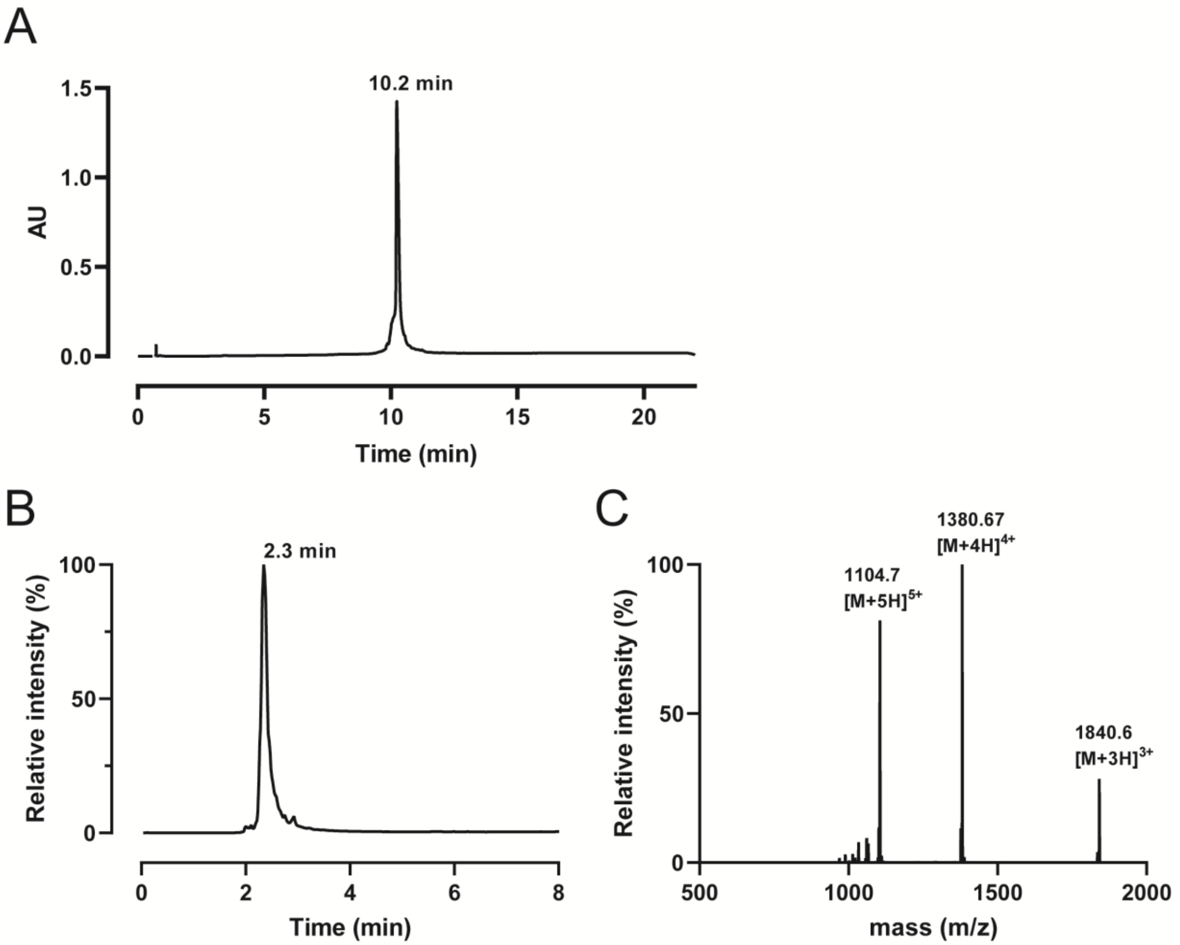

Figure S2. A. UPLC UV chromatogram of purified **2A**, Rt 10.23 min. **B.** Total ion chromatogram (LC-MS method C4) of purified **2A**, Rt 2.35 min. **C.** ESI spectrum of purified **2A**. Calculated Mass (average isotope composition): 5518.53; Observed: $[M + 3H]^{3+}$: 1840.51, $[M + 4H]^{4+}$: 1380.63, $[M + 5H]^{5+}$: 1104.71.

Synthesis of GFP 98-123 propargyl amide

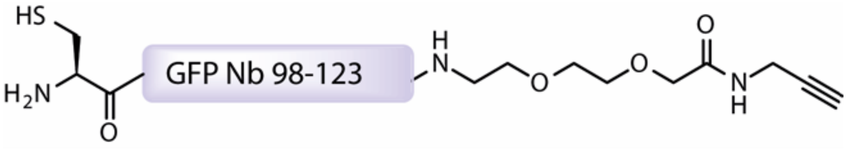

4A

The synthesis was performed following general procedures using Fmoc-OEG preloaded CTC resin (1.45 gram, 0.2 mmol/gram). The amino acids colored in red were coupled using single 6 hours coupling. For the underlined amino acids in the sequence an iso-acyl dipeptide Boc-Thr(Fmoc-Gly)-OH was coupled following the general procedures. The iso-acyl dipeptide was incorporated to increase solubility of the peptide during purification.

CNVNVGFEYWGQGTQVTVSSKHHHHHH

The protected polypeptide was cleaved from the resin by treatment with 3 x 15 mL of DCM/HFIP 7:3 v/v for 15 min and filtered. The combined filtrates were concentrated *in vacuo* and co-evaporated with DCM 3x and dried under high vacuum. The protected peptide (1 equiv.) was dissolved in DCM (10 mL) and propargylamine (75 µL, 1.16 mmol, 4 equiv.) was coupled using PyBOP (597 mg, 1.16 mmol, 4 equiv.) and DIPEA (396 µL, 2.32 mmol, 8 equiv.) for 16 hours. Thereafter, the solvents were removed *in vacuo* and the protecting groups was cleaved according to the general procedures. The crude peptide was purified by RP-HPLC using Phenomenex, Gemini® 110 Å, C18, 5 µm, 30 mm x 250 mm column (15-35 % B over 20 min, flow 30 mL/min) and lyophilized to afford the desired peptide (65.07 mg, 6.7 % yield).

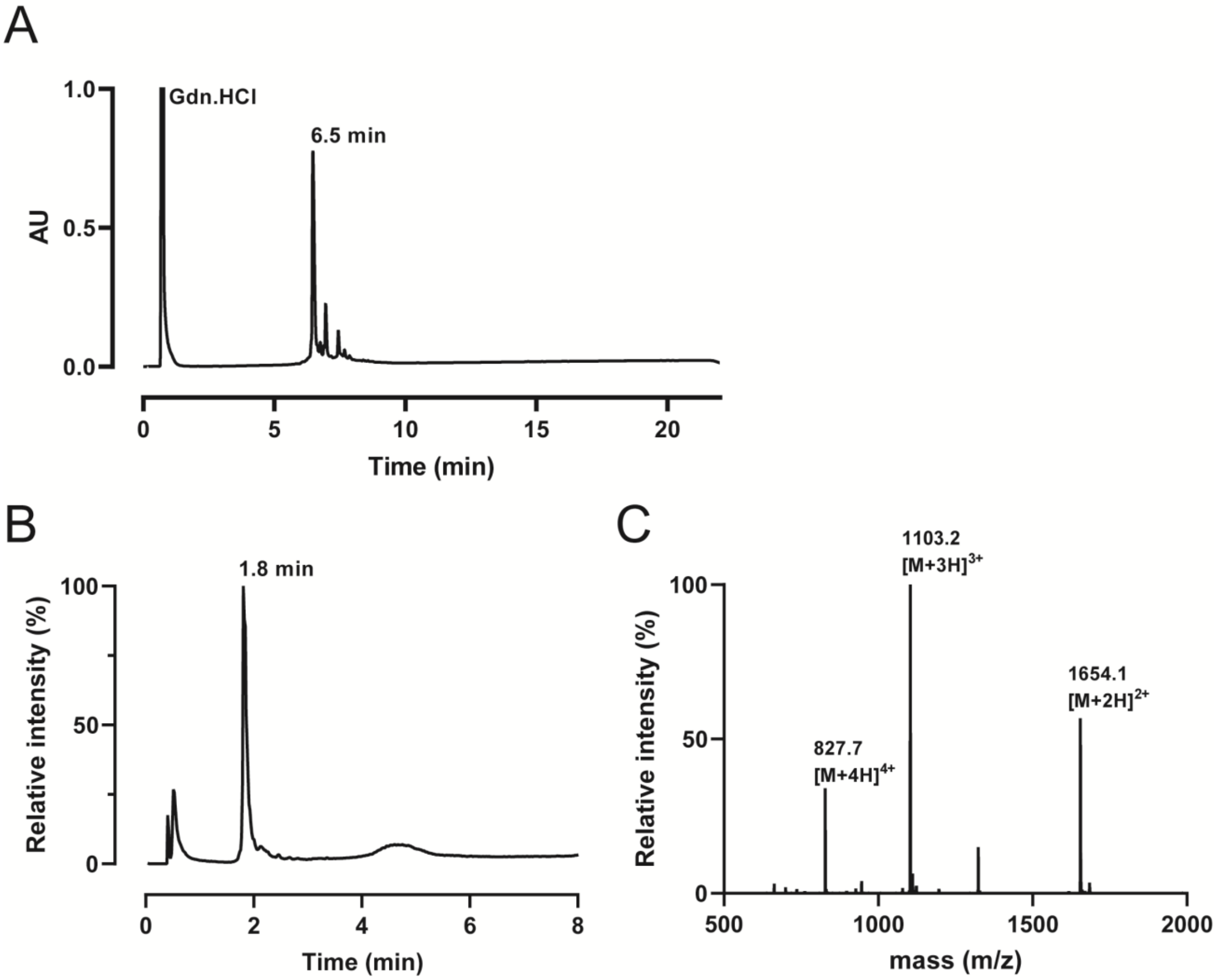

Figure S3. A. UPLC UV chromatogram of purified **4**, Rt 6.53 **B.** Total ion chromatogram (LC-MS method C4) of purified **4**, Rt 1.8 min. **C.** ESI spectrum of purified **4**. Calculated Mass (average isotope composition): 3306.52; Observed: $[M + 2H]^{2+}$: 1654.26, $[M + 3H]^{3+}$: 1103.17, $[M + 4H]^{4+}$: 827.63

Assembly of GFP Nb

One-pot Fmoc deprotection and ligation for the assembly of 3

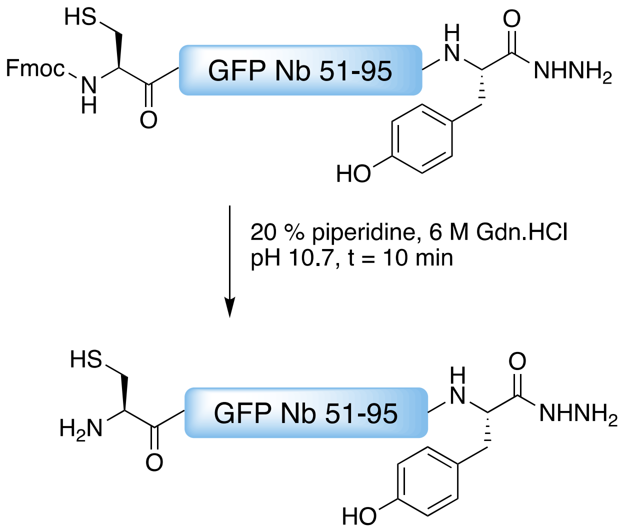

Scheme S2. Fmoc deprotection of **2A**.

A solution of **2A** (73.56 mg, 13.3 µmol) was prepared in 6 M Gdn.HCl, 0.2 M phosphate pH = 7.0 (5 mL), then 406 µL of conc. HCl was added and finally 1375 µL of piperidine, final pH of 10.7. The reaction mixture was shaken (350 rpm) for 10 min before the pH was adjusted to pH 7.0. The reaction progress was assessed by analyzing a small sample by LC-MS. Analysis revealed complete Fmoc deprotection within 10 min. to afford compound **2** in solution.

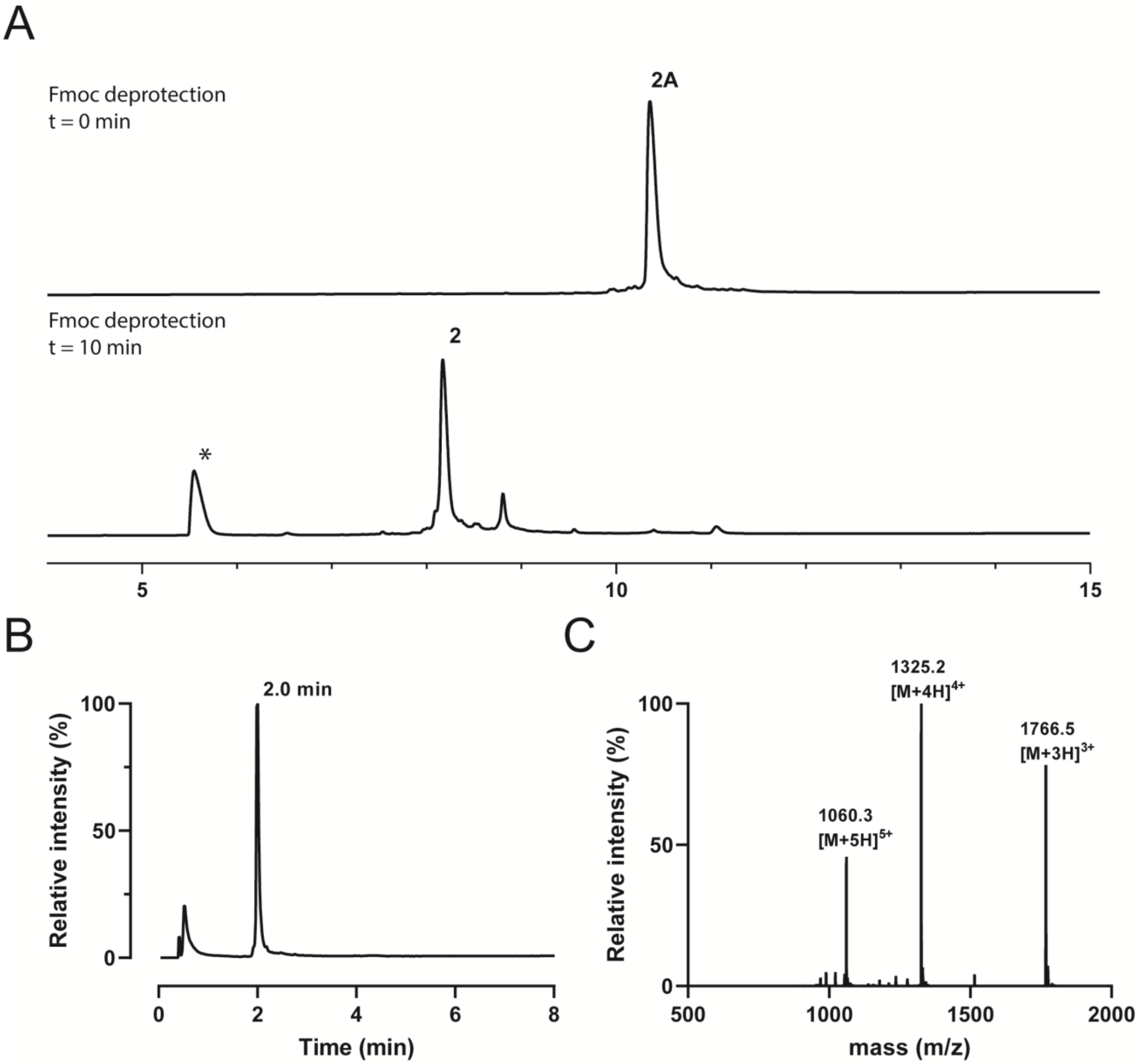

Figure S4. A. UPLC UV chromatogram of the Fmoc deprotection of **2A** t = 0 min. (top chromatogram) and t = 10 min. (bottom chromatogram). *dibenzofulvene-piperidine adduct **B.** Total ion chromatogram (LC-MS method C4) of **2**, Rt 2.0 min. **C.** ESI spectrum of **2**. Calculated Mass (average isotope composition): 5296.46; Observed: $[M + 3H]^{3+}$: 1766.48, $[M + 4H]^{4+}$: 1325.11, $[M + 5H]^{5+}$: 1060.29.

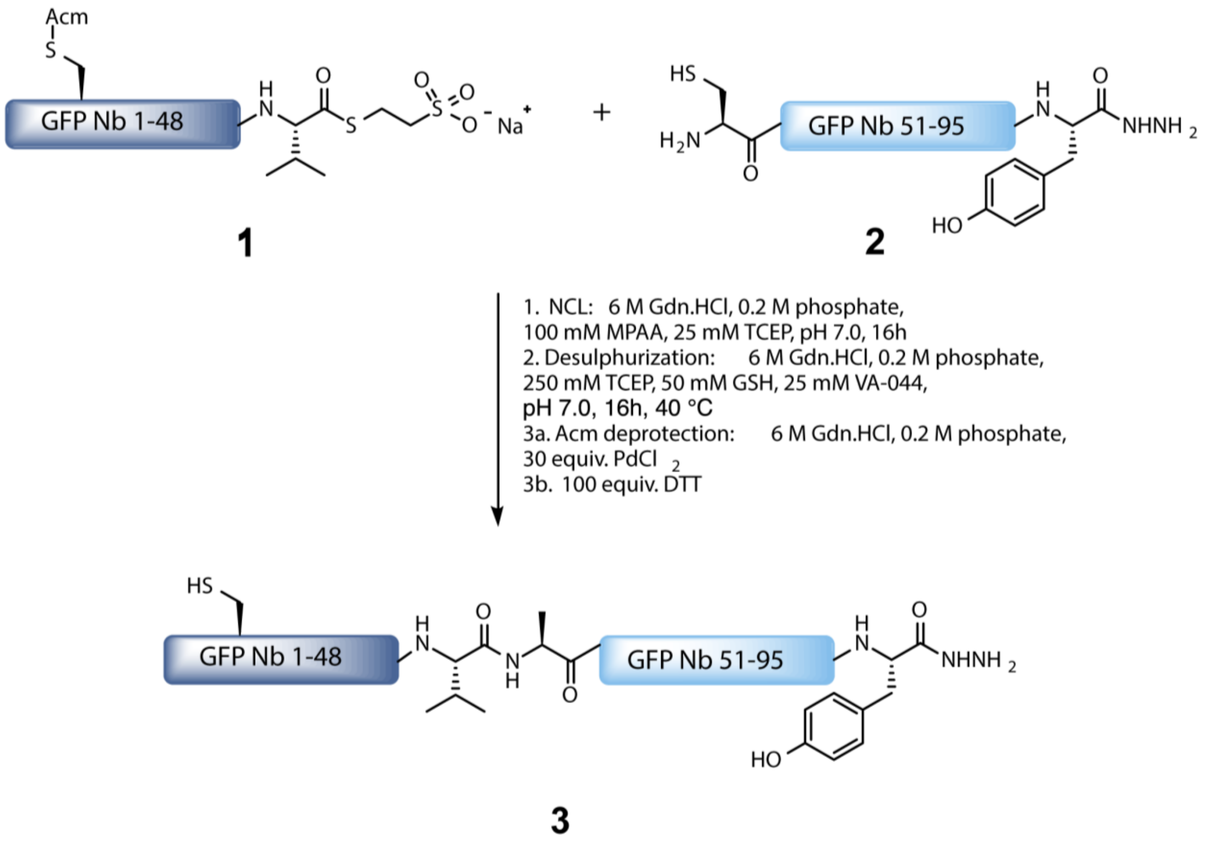

Scheme S3. Ligation of peptides **1** and **2** followed by desulfurization and Acm deprotection to assemble peptide **3**.

Next peptide **1** (60.51 mg, 0.01286 mmol) was added as a solid to the reaction mixture containing **2**. MPAA (186.4 mg, 1.1 mmol, 100 equiv.) was dissolved in 400 µL of 4 M NaOH and added to the reaction mixture and TCEP was added to a final concentration of 25 mM. The final pH was adjusted to 7.0 and the reaction was shaken for 16 hours at 37 °C upon which LCMS analysis showed that the reaction was complete. The MPAA was removed using a 3 kDa cut-off spin filter until LCMS no longer showed presence of MPAA. To the 6 M Gdn.HCl, 0.2 M phosphate pH = 7.0 solution (3 mL), 0.5 M TCEP in water (3 mL) was added and the solution was degassed with argon. To this solution glutathione (101.5 mg, 50 mM) and VA-044 (57 mg, 25 mM) were added and the reaction mixture was shaken at 40 °C for 18 hours at final pH 6.5. To afford peptide **3** in 101.6 mg with a 88 % yield over 2 steps. The buffer was exchanged again using a 3 kDa cut-off spin filter to 6 M Gdn.HCl, 0.2 M phosphate pH 7.0. Next $PdCl_2$ (29.6 mg, 20 equiv.) was added, and the reaction was shaken at 40 °C for 1 hour. To quench the reaction DTT (137.3 mg, 100 equiv.) was added and the solution was centrifuged. The supernatant was purified on an Äkta system using a HiLoad® 26/600 Superdex® 75 pg column (flow: 1 mL/min) to obtain GFP 1-96 in a solution of 6 M Gdn.HCl, 0.2 M phosphate, pH 7.0 (9.6 mg/mL, 62.2 mg, 61.7 % yield over 4 steps).

All concentrations/amounts were determined using CAD as described in the general protocols.

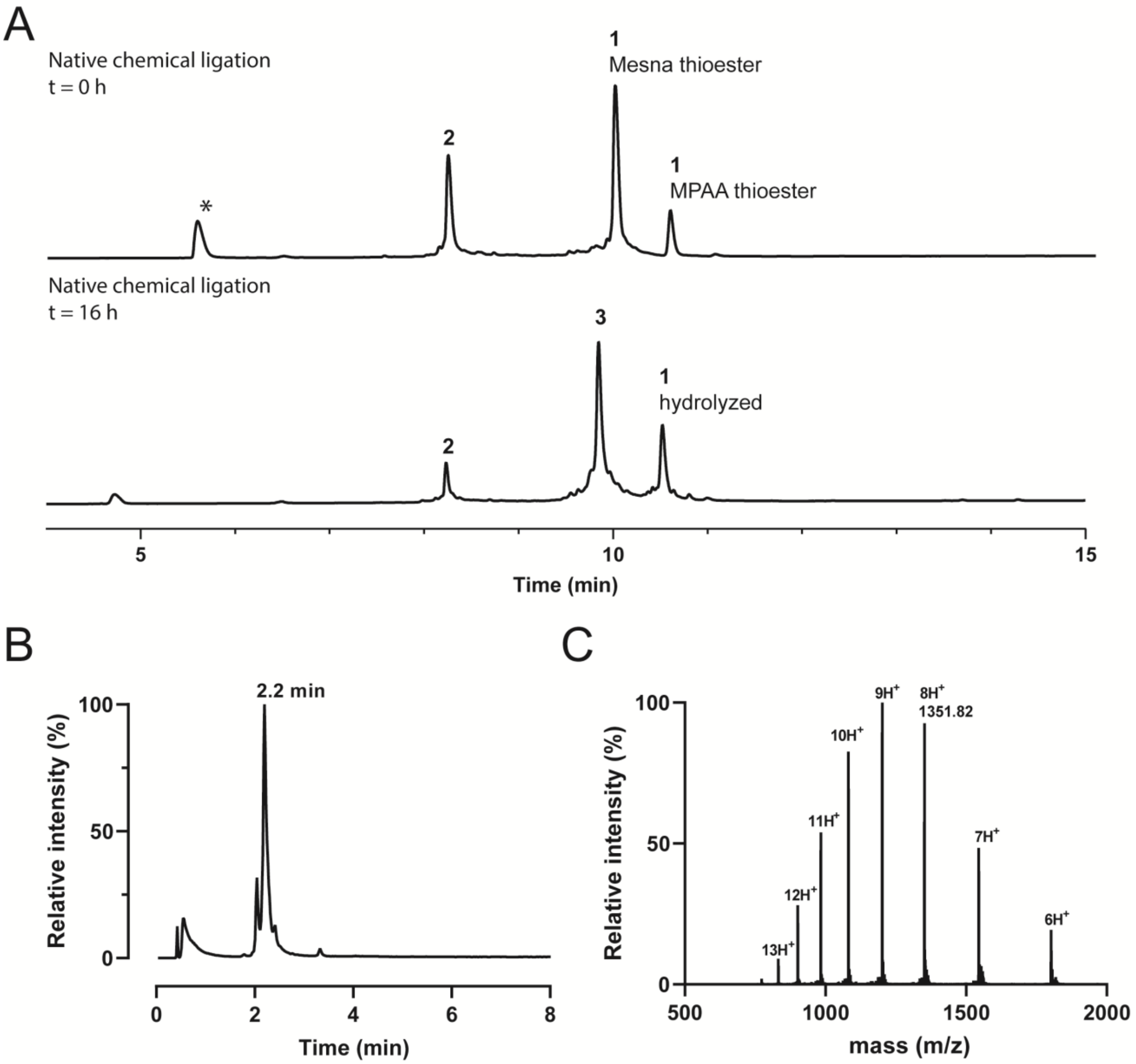

Figure S5. A. UPLC UV chromatogram of the NCL of **1** and **2**, t = 0 h (top chromatogram) and t = 16 h (bottom chromatogram). *dibenzofulvene-piperidine adduct **B.** Total ion chromatogram (LC-MS method C4) of **3**, Rt 2.2 min. **C.** ESI spectrum of **3**. Calculated Mass (average isotope composition): 10800.22; $[M + 6H]^{6+}$: 1801.04, $[M + 7H]^{7+}$: 1543.89, $[M + 8H]^{8+}$: 1351.03, $[M + 9H]^{9+}$: 1201.02, $[M + 10H]^{10+}$: 1081.02, $[M + 11H]^{11+}$: 982.84, $[M + 12H]^{12+}$: 901.08, $[M + 13H]^{13+}$: 831.77. Observed Mass (average isotope composition): 10801.52; $[M + 6H]^{6+}$: 1801.25, $[M + 7H]^{7+}$: 1544.08, $[M + 8H]^{8+}$: 1351.82, $[M + 9H]^{9+}$: 1201.17, $[M + 10H]^{10+}$: 1081.15, $[M + 11H]^{11+}$: 982.96, $[M + 12H]^{12+}$: 901.12, $[M + 13H]^{13+}$: 831.88.

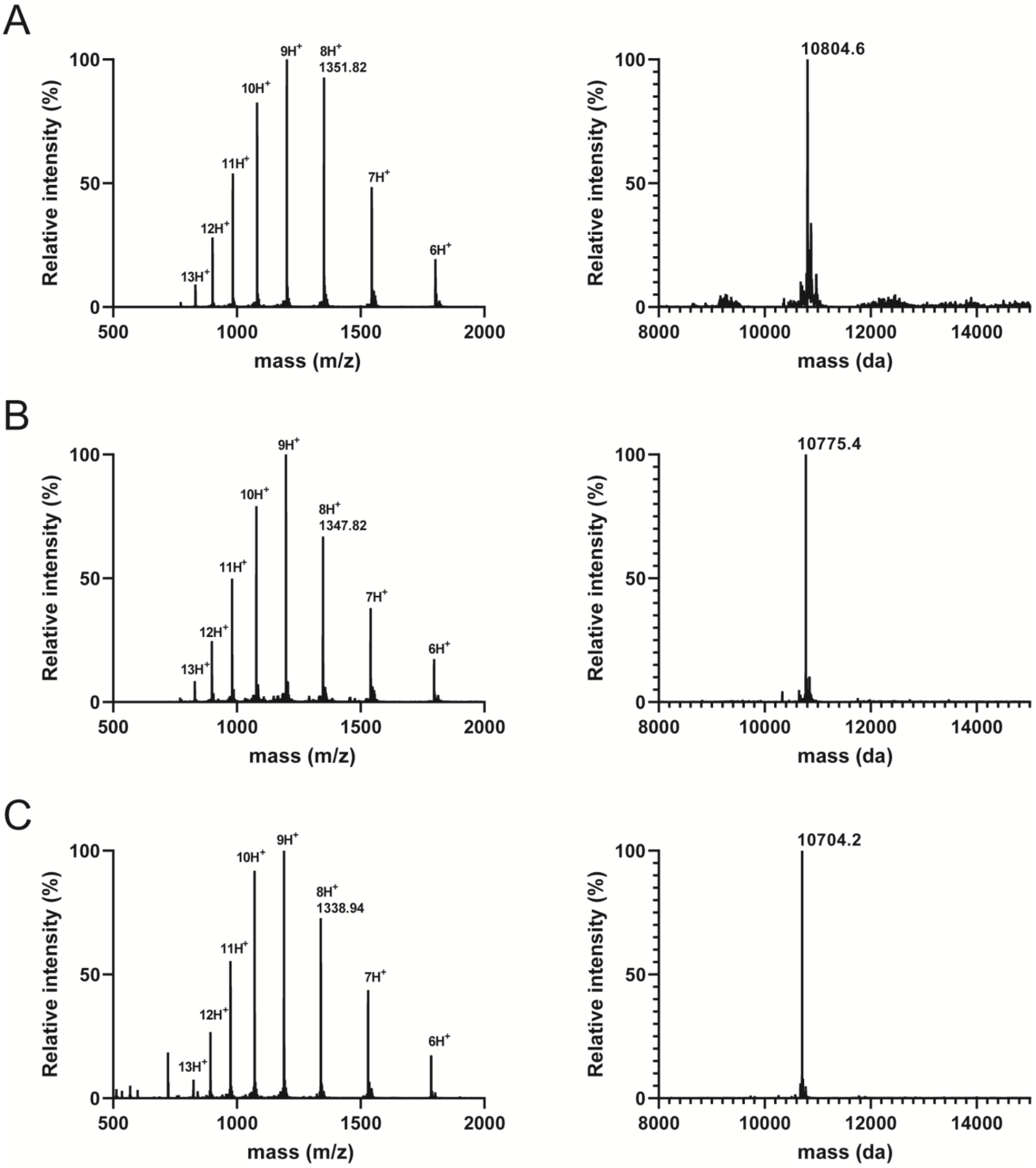

Figure S6. A. ESI spectrum and the deconvoluted mass of **3A**. Calculated Mass (average isotope composition):10800.22, Observed: 10801.52. Deconvoluted mass calculated: 10807.0, Observed: 10804.6 **B.** ESI spectrum and the deconvoluted mass of **3B**. Calculated Mass (average isotope composition): 10768.25, Observed: 10769.52. Deconvolute mas calculated: 10774.9, Observed: 10775.4. **C.** ESI spectrum and the deconvoluted mass of **3** Calculated Mass (average isotope composition): 10697.21, Observed: 10698.4. Deconvolute mass calculated: 10703.9, Observed: 10704.2.

Iso-acyl shift of 4A

As described in the synthesis section of peptide **4A**, an iso-acyl dipeptide was incorporated to increase solubility during purification.[8–10] The ester bond is not stable during NCL and therefore has to undergo an O → N acyl shift to form the stable native peptide (Scheme S4).

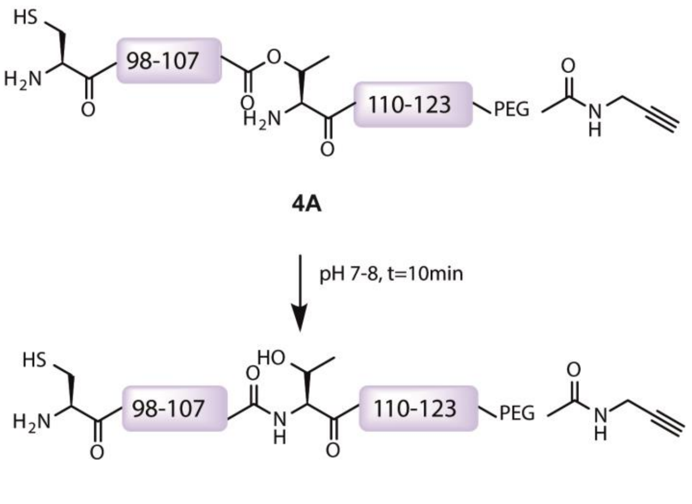

Scheme S4. Shift of the iso-acyl dipeptide.

The peptide **4A** (9.4 mg, 2.8 μmol) was dissolved in 400 μL 6 M Gdn.HCl, 0.2 M phosphate, pH 7.4. After 10 minutes an UPLC sample was measured and the retention time of the peptide shifted from 6.47 to 6.96 minutes (UPLC method 2), indicating that the iso-acyl had shifted successfully (Fig. S7).

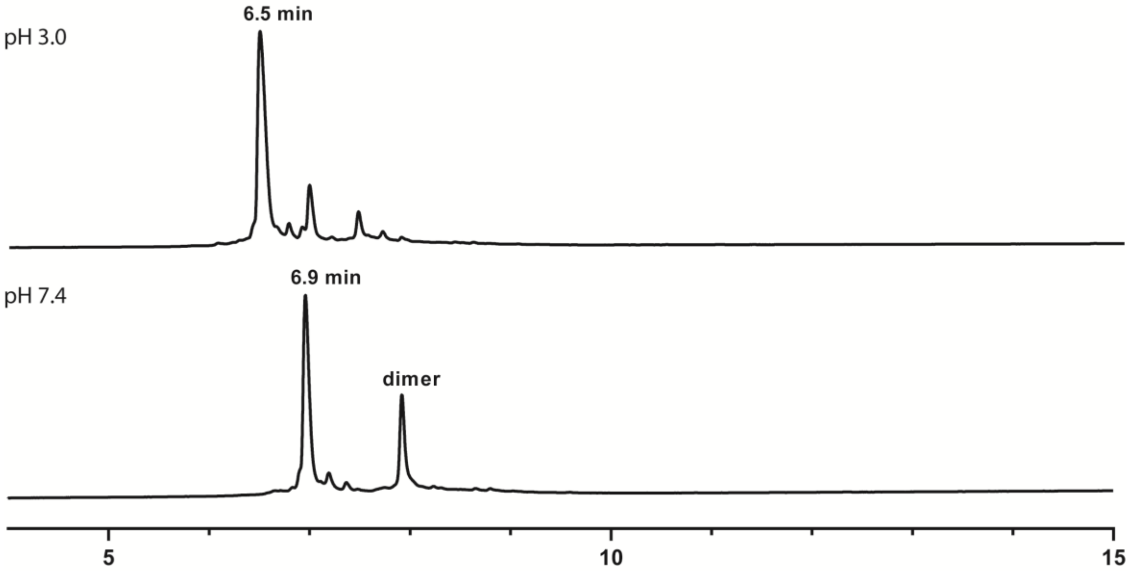

Figure S7. UPLC UV chromatogram of **4A** at pH 3.0 (top chromatogram) and **4** at pH 7.4 (bottom chromatogram).

One-pot thioesterification and ligation to GFP 1-123-PA

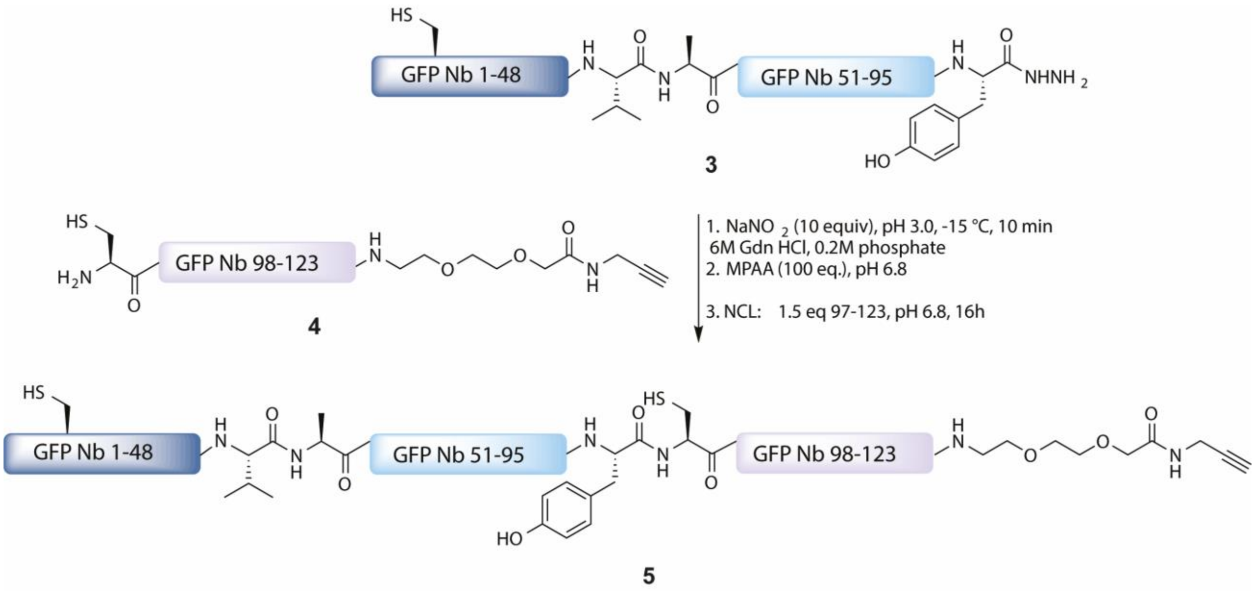

Scheme S5. One-pot thioesterification and NCL of **3** and **4** to obtain the final product **5**.

A solution of **3** (28.8 mg, 2.69 μmol) in 3 mL of 6 M Gdn.HCl, 0.2 M phosphate, pH 3.0 was cooled to 0 °C before adding 27 μL of 1 M $NaNO_2$ in MilliQ. After 15 minutes the solution was warmed to room temperature and MPAA (51 mg, 303 μmol, 100 equiv.) in 4 M NaOH (50 μL), and **4** (9.4 mg, 2.8 μmol) were added, and the pH was adjusted to pH 7.13. The mixture was shaken over night at room temperature to reach completion before purification on a Äkta system using a HiLoad® 26/600 Superdex® 75 pg column (flow: 1 mL/min) to obtain in a solution of 6 M Gdn.HCl, 0.2 M phosphate, pH 7.0 (9.13 mg/mL, 13.71 mg, 36.4 % yield based on recovered starting material).

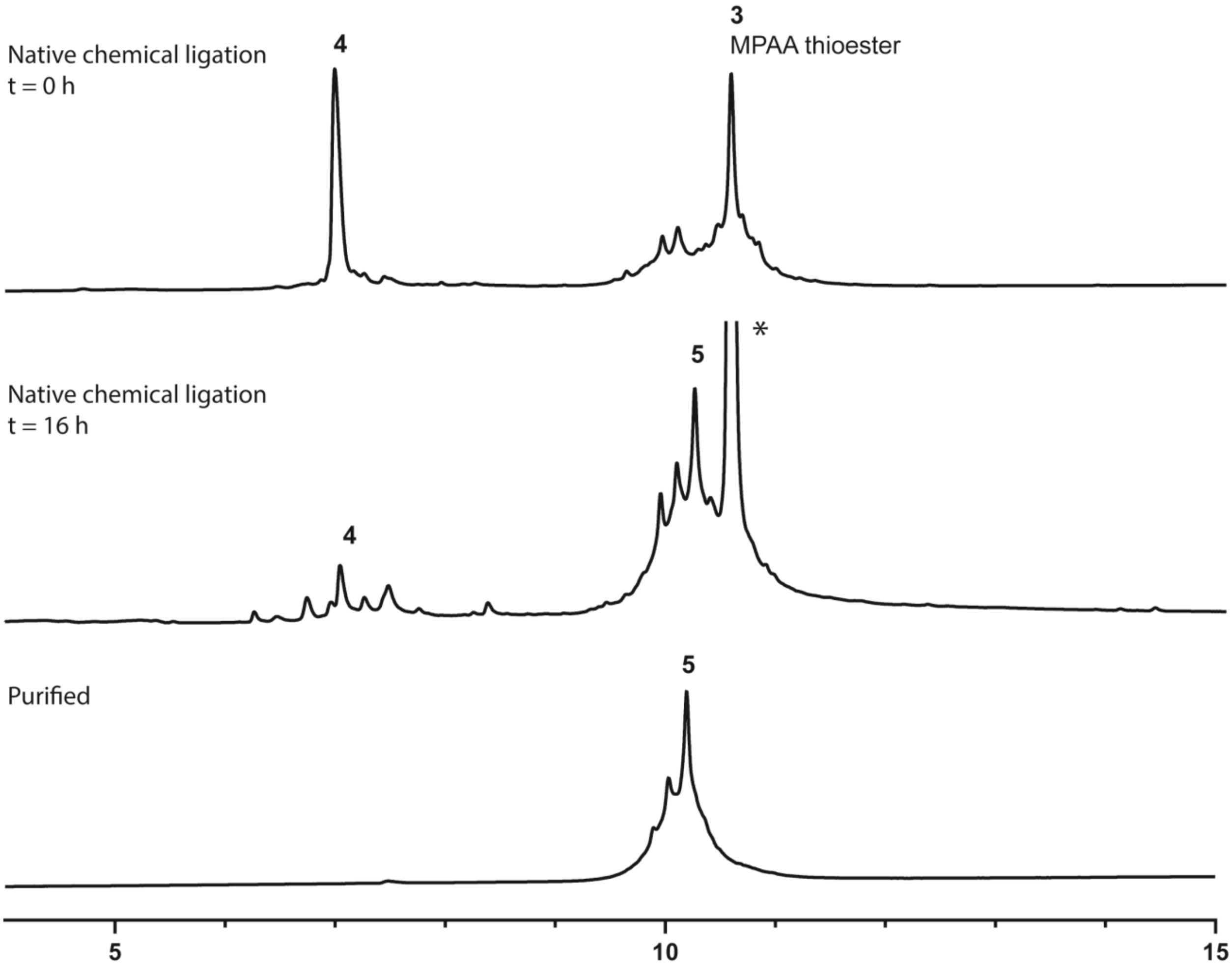

Figure S8. UPLC UV chromatogram of NCL between **4** and **3** t = 0 h (top chromatogram), t = 16 h (middle chromatogram), and purified **5** (bottom chromatogram)

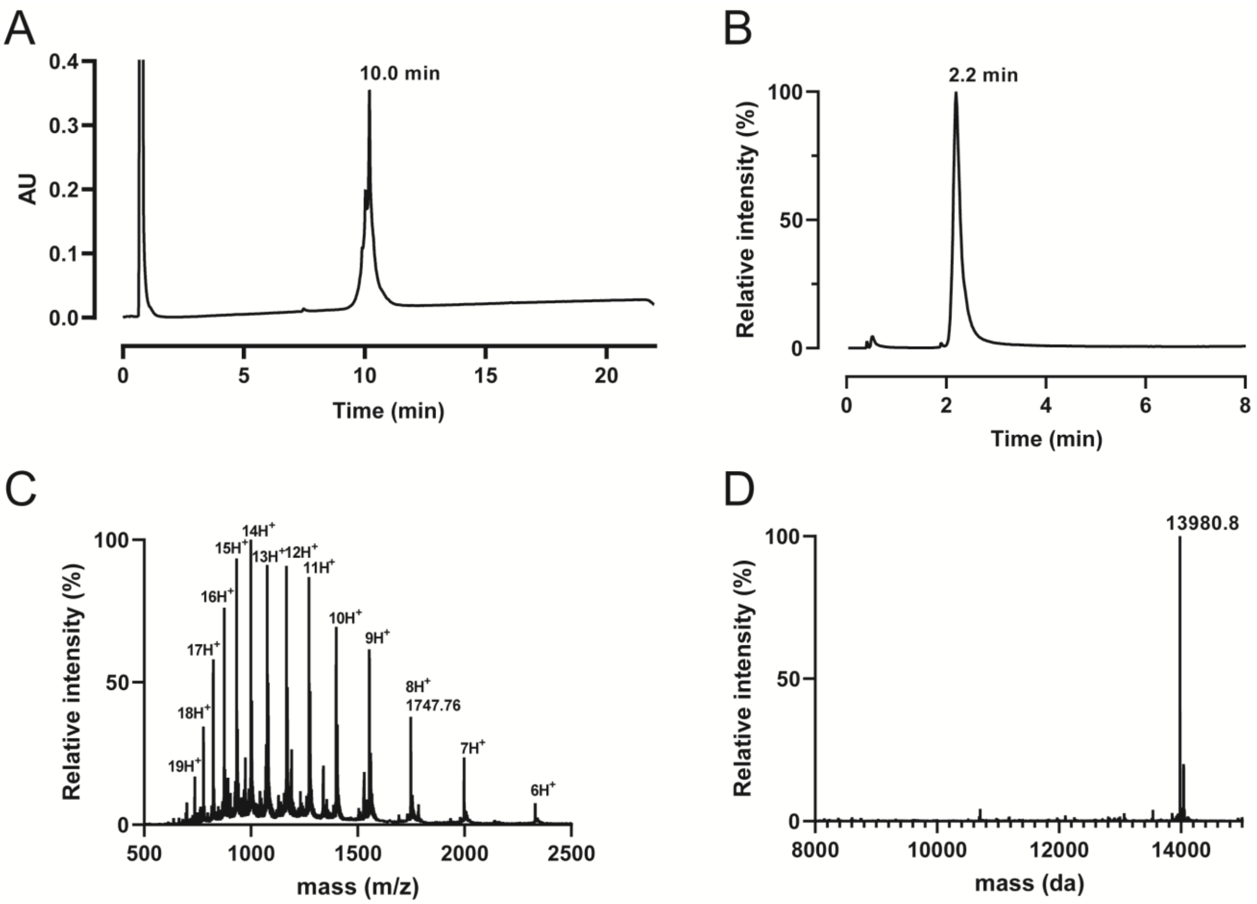

Figure S9. A. UPLC UV chromatogram of **5**, Rt 10.0 min. **B.** Total ion chromatogram (LC-MS method C4) of **5**, Rt 2.2 min min. **C.** ESI spectrum of **5**. Calculated Mass (average isotope composition): 13971.70; [M + 6H]$^{6+}$: 2329.62, [M + 7H]$^{7+}$: 1996.95, [M + 8H]$^{8+}$: 1747.46, [M + 9H]$^{9+}$: 1553.41, [M + 10H]$^{10+}$: 1398.17, [M + 11H]$^{11+}$: 1271.16, [M + 12H]$^{12+}$: 1165.31, [M + 13H]$^{13+}$: 1075.75, [M + 14H]$^{14+}$: 998.97. Observed: ; [M + 6H]$^{6+}$: 2329.81, [M + 7H]$^{7+}$: 1997.14, [M + 8H]$^{8+}$: 1747.62, [M + 9H]$^{9+}$: 1553.45, [M + 10H]$^{10+}$: 1398.30, [M + 11H]$^{11+}$: 1271.18, [M + 12H]$^{12+}$: 1165.33, [M + 13H]$^{13+}$: 1075.78, [M + 14H]$^{14+}$: 999.00. **D.** Deconvoluted mass of **5**, calculated: 13980.4, observed: 13980.8.

Folding of 5

PBS buffer, pH 7.4 was freshly prepared from Gibco PBS tablets and sterilized with a bottle top vacuum filter, 0.22 µm (Corning). A solution of crude unfolded **5** (0.65 mM, 1.5 mL) in 6 M Gdn.HCl, 0.2 M phosphate, pH 7.0 was added to a prewashed Slide-A-Lyzer™ MINI Dialysis Devices (3.5 kDa cut-off) containing 3 M Gdn.HCl 0.2 M phosphate, pH 7.0. After 2 hours the buffer was exchanged to PBS, pH 7.4 and the mixture was shaken gently over night at 10 °C. The mixture was analyzed by LC-MS revealing the correct MW corresponding to a loss of 2 Da. The mixture was concentrated using a pre-washed centrifugal 3 kDa molecular weight cut-off device and concentrated to ~ 400 µL.

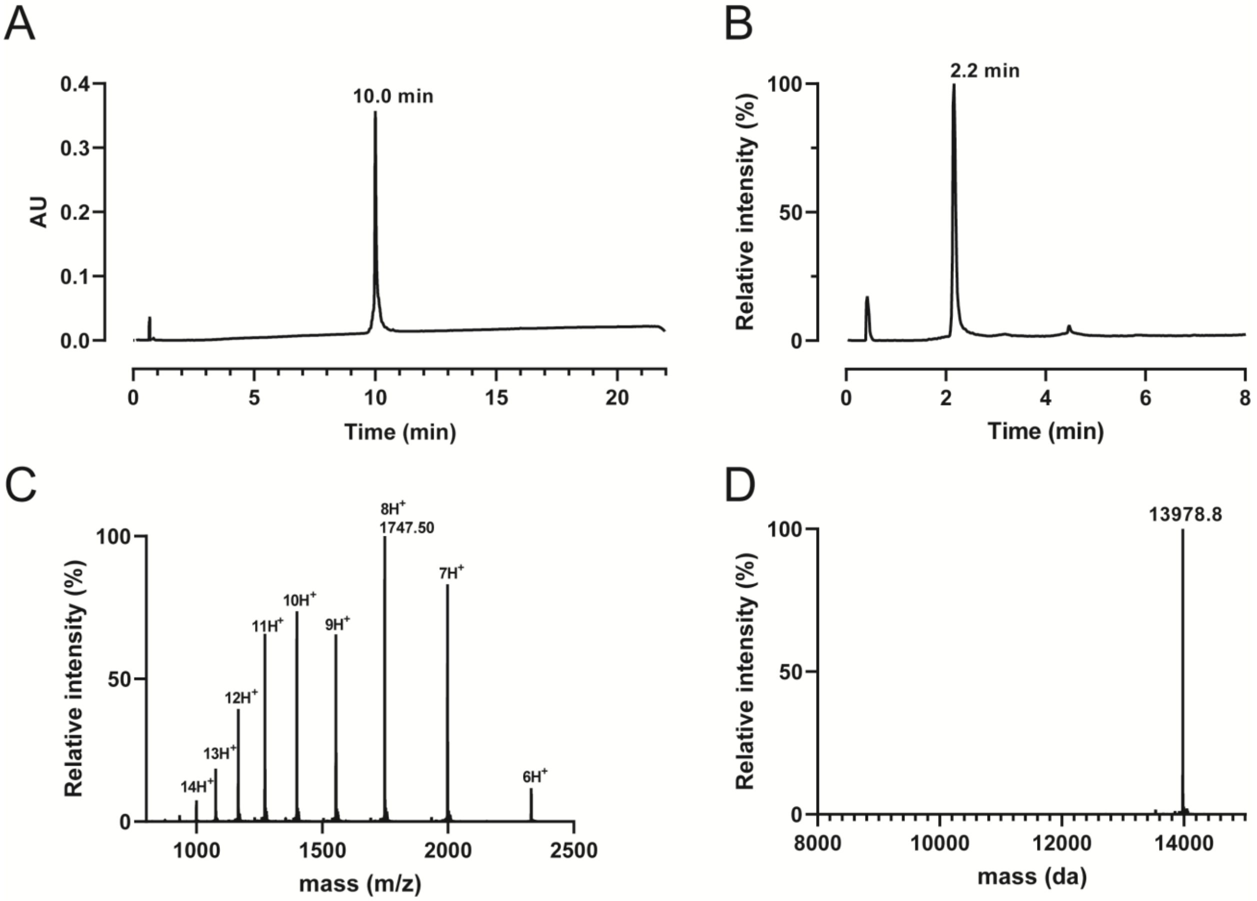

Figure S10. A. UPLC UV chromatogram (method 1) of folded **5**, Rt 10.0 min. **B.** Total ion chromatogram (LC-MS method C4) of folded **5**, Rt 2.2 min **C.** ESI spectrum of folded **5.** Calculated Mass (average isotope composition): 13969.68; $[M + 6H]^{6+}$: 2329.28, $[M + 7H]^{7+}$: 1996.70, $[M + 8H]^{8+}$: 1747.21, $[M + 9H]^{9+}$: 1553.19, $[M + 10H]^{10+}$: 1398.97, $[M + 11H]^{11+}$: 1270.97, $[M + 12H]^{12+}$: 1165.14, $[M + 13H]^{13+}$: 1075.59, $[M + 14H]^{14+}$: 998.83. Observed: 13973.0; $[M + 6H]^{6+}$: 2329.83, $[M + 7H]^{7+}$: 1997.14, $[M + 8H]^{8+}$: 1747.50, $[M + 9H]^{9+}$: 1553.45, $[M + 10H]^{10+}$: 1398.30, $[M + 11H]^{11+}$: 1271.18, $[M + 12H]^{12+}$: 1165.33, $[M + 13H]^{13+}$: 1075.78, $[M + 14H]^{14+}$: 999.00. **D.** Deconvoluted mass of folded **5**, calculated: 13978.4, Observed: 13978.8.

CuAAC chemistry on 5

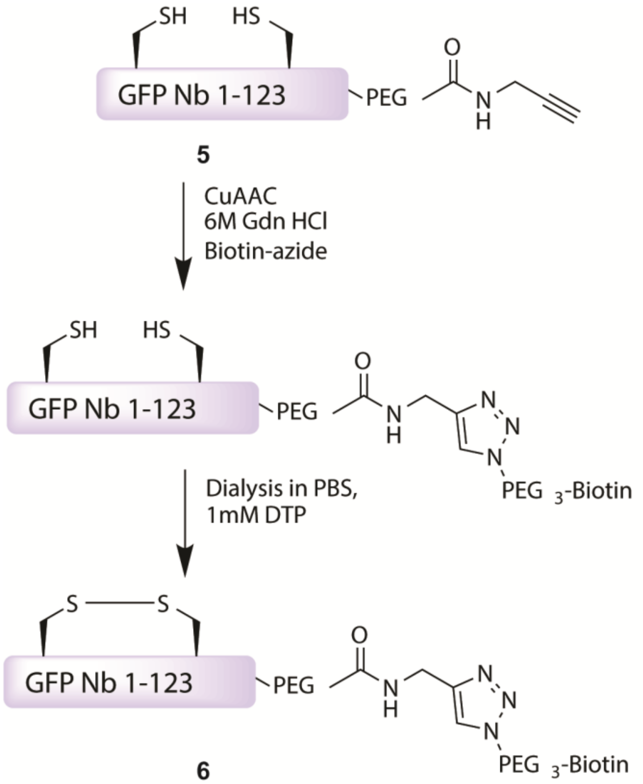

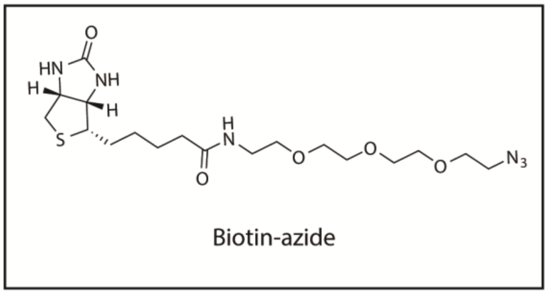

Scheme S6. Click chemistry on purified **5** followed by disulfide bond formation of **6**.

To purified **5** (3.65 mg, 0.26 μmol, 1.0 equiv.) in 400 μL 6 M Gdn.HCl, 0.2 M phosphate pH 7.2, 25 μL of freshly prepared click-mixture (1:1:1 v/v/v, $CuSO_4{\cdot}5H_20$ (40.7 mg/mL in water): sodium ascorbate (120 mg/mL in water): THPTA ligand (42.5 mg/mL in water)) was added before adding 65 μL of Biotin-PEG-azide (CAS Number: 875770-34-6)(10 mM in DMSO, 0.65 μmol, 2.5 equiv.). The reaction was shaken for 60 minutes at room temperature when LC-MS showed full conversion to **6**. The reaction mixture was quenched with 5 μL EDTA (0.5 M in MilliQ water) before purification by Äkta, Superdex® 200 Increase 10/300 GL (flow: 0.5 mL/min) to obtain **6** (2.48 mg, 66 % yield). Thereafter, **6** was folded as previously described for **5**, resulting in folded **6** (Fig S11).

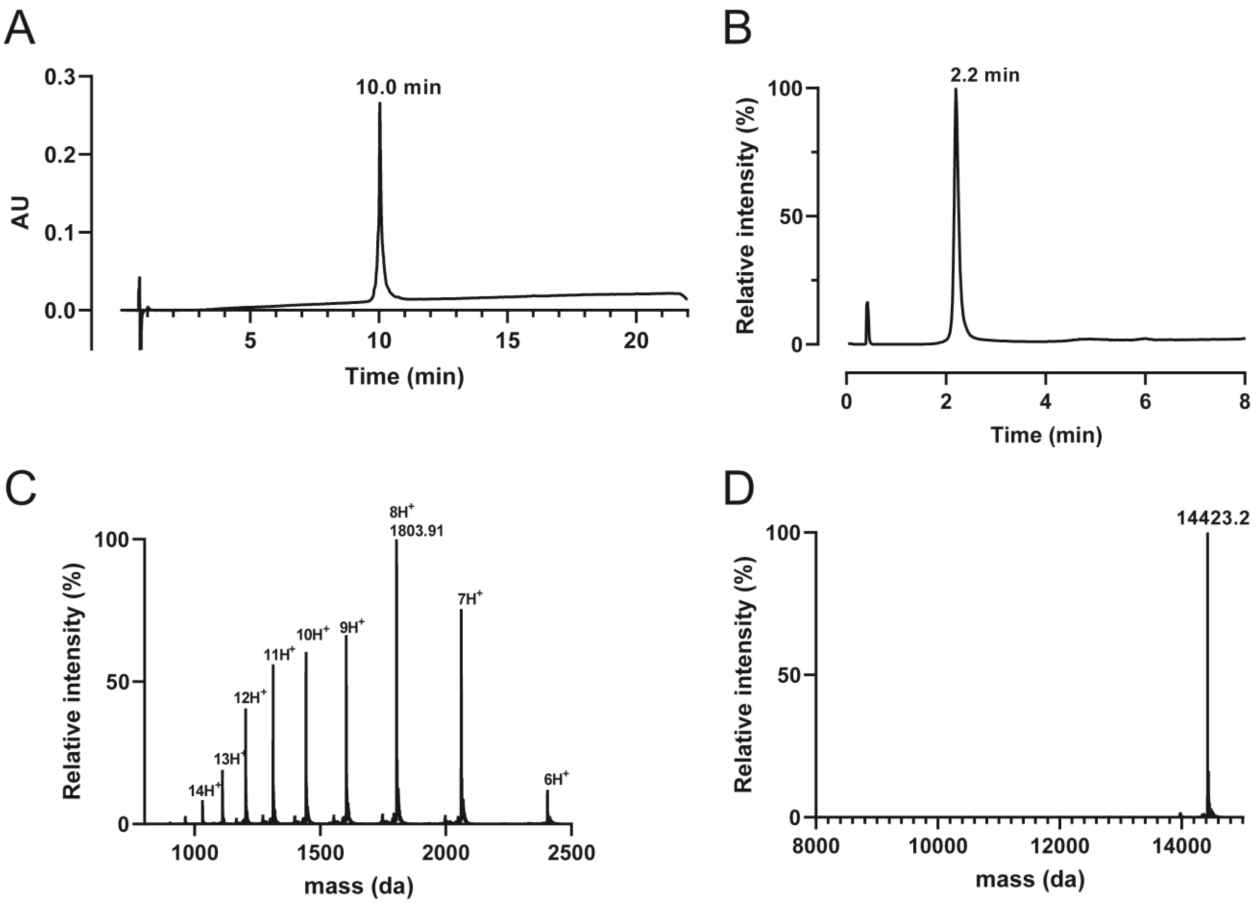

Figure S11. A. UPLC UV chromatogram of folded **6**, Rt 10.0 min. **B.** Total ion chromatogram (LC-MS method C4) of folded **6**, Rt 2.2 min **C.** ESI spectrum of folded **6**. Calculated Mass (average isotope composition): 14422.9; $[M + 6H]^{6+}$: 2403.32, $[M + 7H]^{7+}$: 2060.13, $[M + 8H]^{8+}$: 1802.74, $[M + 9H]^{9+}$: 1602.54, $[M + 10H]^{10+}$: 1442.39, $[M + 11H]^{11+}$: 1311.35, $[M + 12H]^{12+}$: 1202.16, $[M + 13H]^{13+}$: 1109.76, $[M + 14H]^{14+}$: 1030.56, $[M + 15H]^{15+}$: 961.93. Observed: Calculated Mass (average isotope composition): 14423.2.4; $[M + 6H]^{6+}$: 2403.67, $[M + 7H]^{7+}$: 2060.46, $[M + 8H]^{8+}$: 1803.16, $[M + 9H]^{9+}$: 1602.80, $[M + 10H]^{10+}$: 1442.63, $[M + 11H]^{11+}$: 1311.56, $[M + 12H]^{12+}$: 1202.35, $[M + 13H]^{13+}$: 1109.95, $[M + 14H]^{14+}$: 1030.73. **D.** Deconvoluted mass of folded **6**, calculated: 14422.8, observed: 14423.2.

Click chemistry on 5

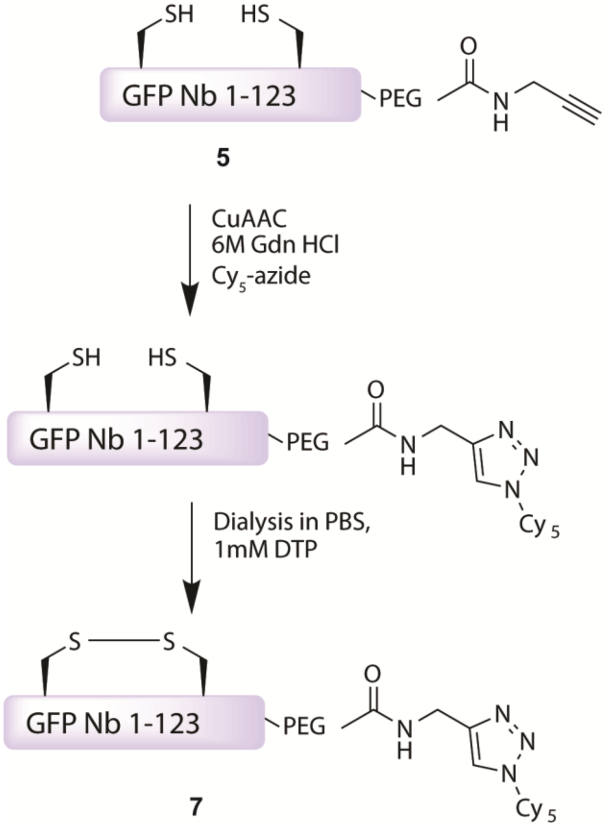

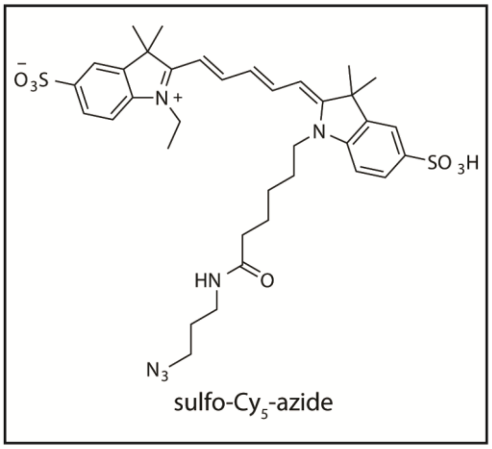

Scheme S7. Click chemistry on purified **5** followed by disulfide bond formation of **7**.

To purified **5** (3.65 mg, 0.26 µmol, 1.0 equiv.) in 400 µL 6 M Gdn.HCl, 0.2 M phosphate pH 7.2, 25 µL of freshly prepared click-mixture (1:1:1 v/v/v, $CuSO_4 \cdot 5H_20$ (40.7 mg/mL in water): sodium ascorbate (120 mg/mL in water): THPTA ligand (42.5 mg/mL in water)) was added before adding 65 µL of sulfo-Cy5-azide (CAS Number. : 1621101-43-6) (10 mM in DMSO, 0.65 µmol, 2.5 equiv.). The reaction was shaken for 60 minutes at room temperature when LC-MS showed full conversion of the **7**. The reaction mixture was quenched with 5 µL EDTA (0.5M in MilliQ water) before purification by Äkta, Superdex® 200 Increase 10/300 GL (flow: 0.5 mL/min) to obtain **7** (1.58 mg, 41 % yield). Thereafter, **7** was folded as previously described for **5**, resulting in folded **7** (Fig S?.).

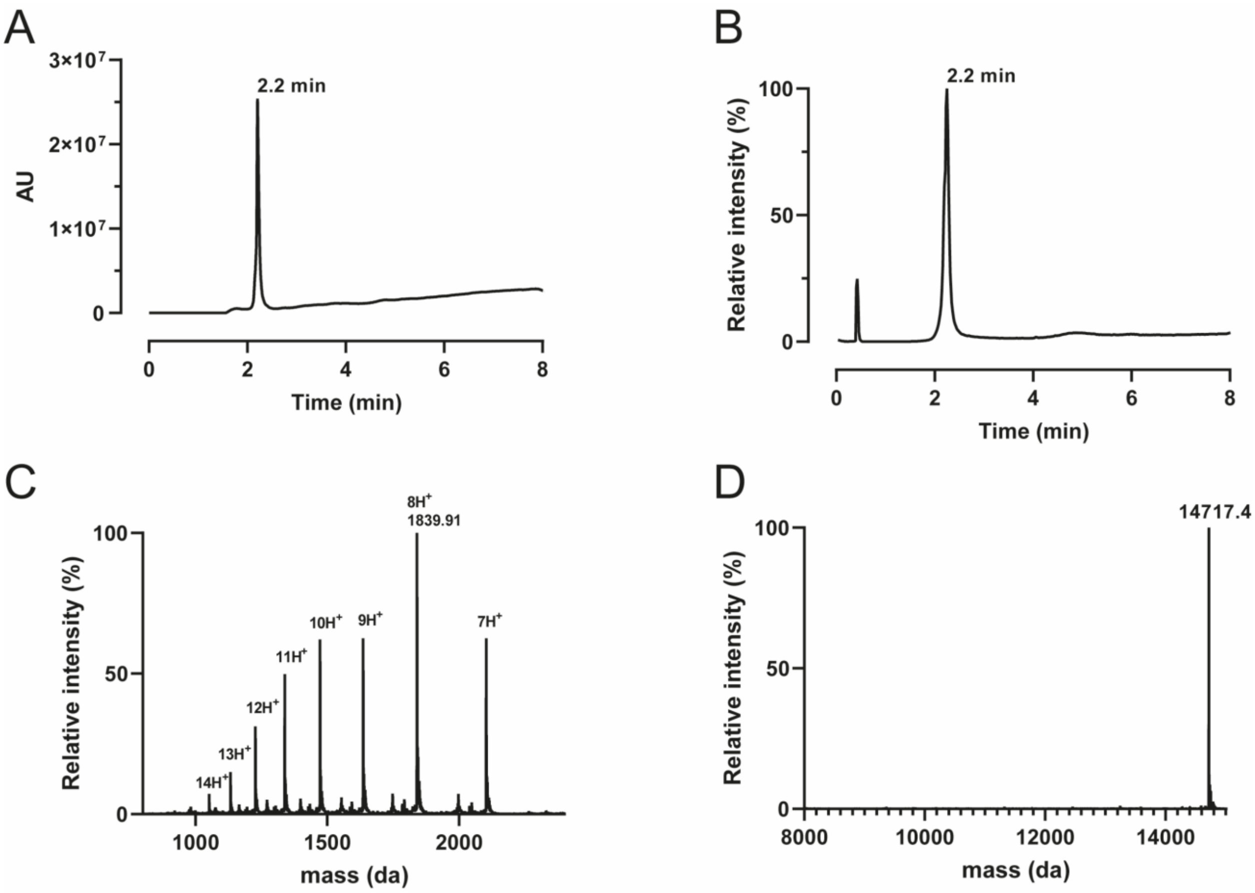

Figure S12. A. UPLC UV chromatogram of folded **7**, Rt 10.0 min. **B.** Total ion chromatogram (LC-MS method C4) of folded **7**, Rt 2.2 min **C.** ESI spectrum of folded **7.** Calculated Mass (average isotope composition): 14717.3; $[M + 6H]^{6+}$: 2452.33, $[M + 7H]^{7+}$: 2102.14, $[M + 8H]^{8+}$: 1839.50, $[M + 9H]^{9+}$: 1635.22, $[M + 10H]^{10+}$: 1471.80, $[M + 11H]^{11+}$: 1338.09, $[M + 12H]^{12+}$: 1226.66, $[M + 13H]^{13+}$: 1132.38, $[M + 14H]^{14+}$: 1051.56. Observed: Calculated Mass (average isotope composition): 14717.4; $[M + 6H]^{6+}$: 2452.86, $[M + 7H]^{7+}$: 2102.62, $[M + 8H]^{8+}$: 1839.78, $[M + 9H]^{9+}$: 1635.59, $[M + 10H]^{10+}$: 1472.04, $[M + 11H]^{11+}$: 1338.31, $[M + 12H]^{12+}$: 1226.87, $[M + 13H]^{13+}$: 1132.57, $[M + 14H]^{14+}$: 1051.83. **D.** Deconvuluted mass of folded **7**, calculated: 14717.3 Observed: 14717.4.

Circular dichroism

CD measurements were performed using a Jasco 1500 spectropolarimeter at concentrations of 0.1 mg/mL in PBS, pH 7.4, concentrations were measured using a NanoDrop spectrophotometer at A280 (calculated extinction coefficient of 26930 $cm^{-1}M^{-1}$). Measurements between 250 and 190 nm were taken using a quartz cuvette with a path length of 0.02 cm. In total, 8 cumulative measurements were made and the average was calculated and plotted using Graphpad PRISM.

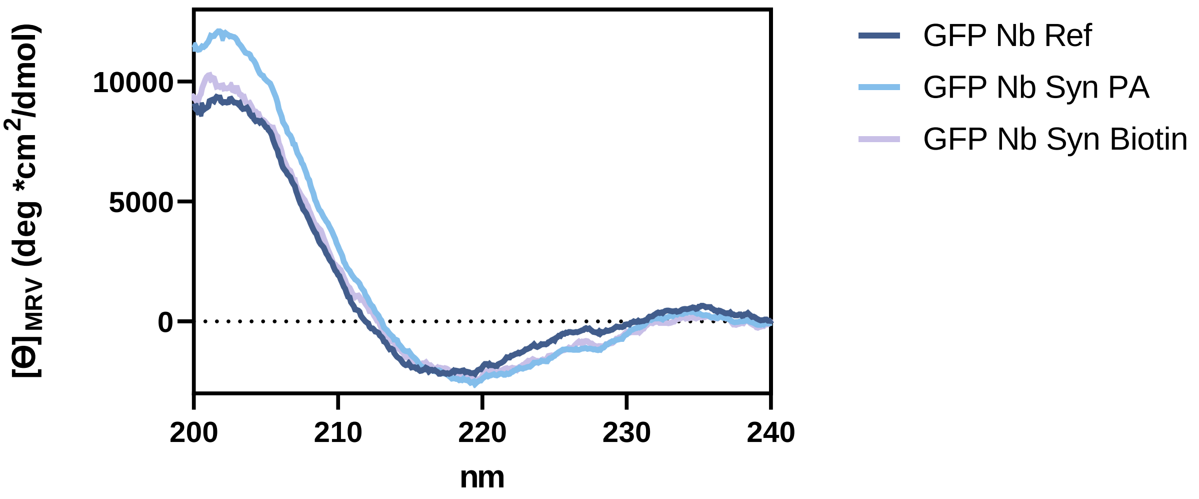

Figure S13. CD chromatogram of the expressed Nb, **5** and **6**.

Unfolding CD measurements were performed with a 1 °C/min increase, with a measurement containing 8 scans every 10 °C from 20 °C to 90 °C.

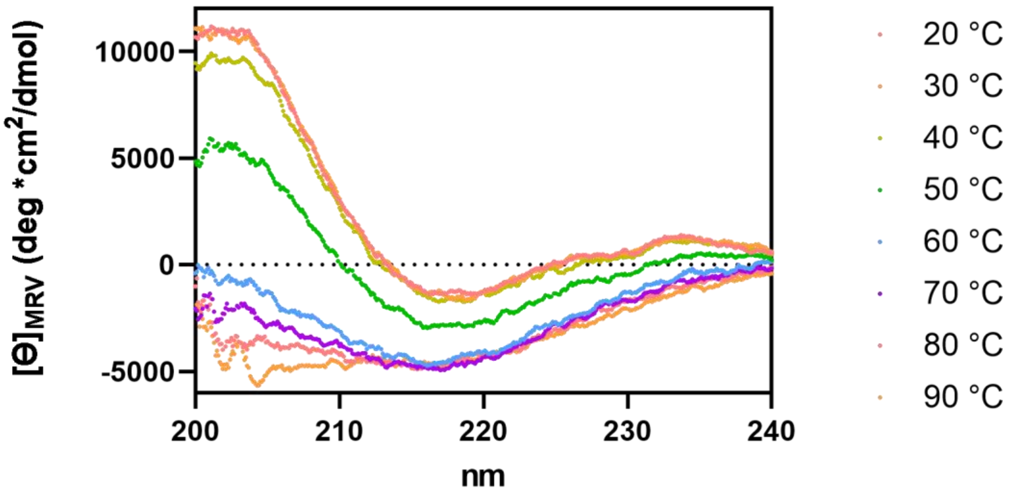

Figure S14. CD spectra of expressed GFP Nb with heating.

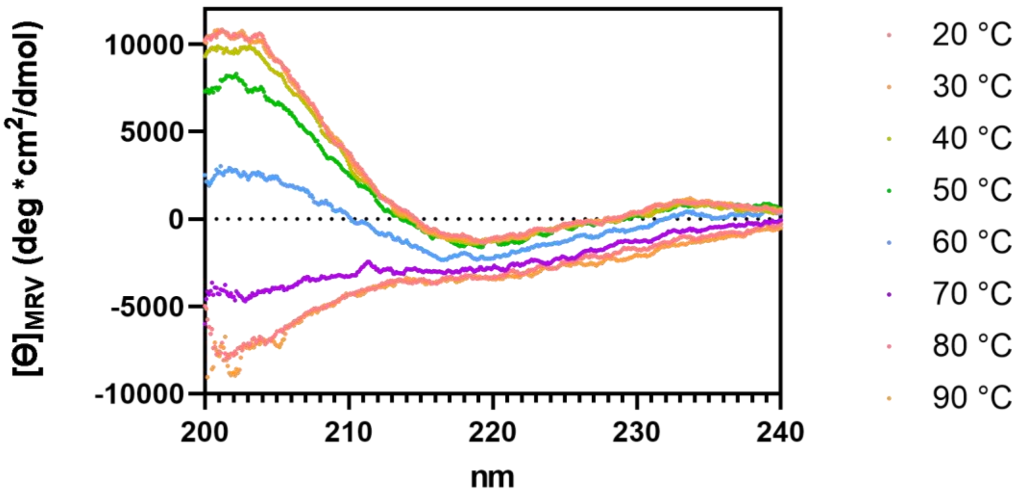

Figure S15. CD spectra of **6** with heating.

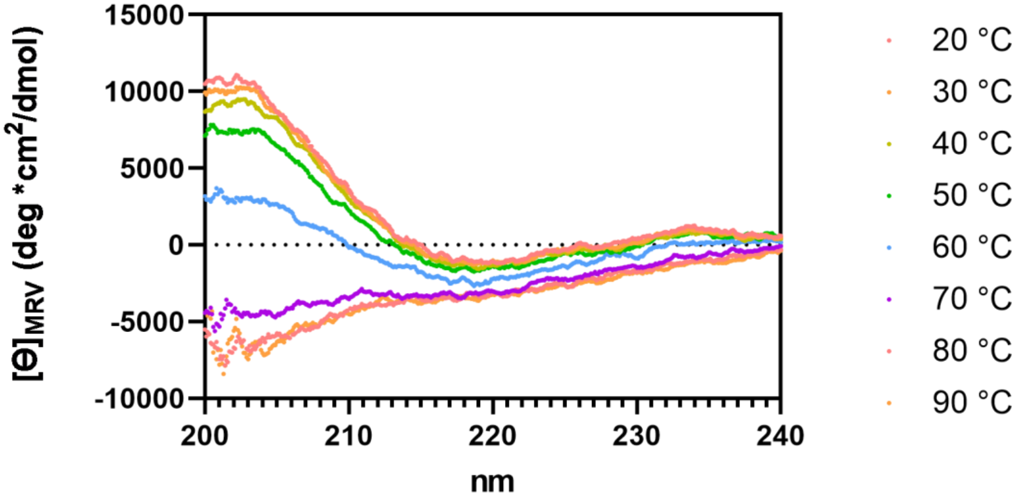

Figure S16. CD spectra of **7** with heating.

Bio Layer Interferometry

Bio Layer Interferometry (BLI) analyses of binding experiments. Graphs show concentrations in nM and fitted curves as dotted lines. The data was fitted using the Octet96 software.

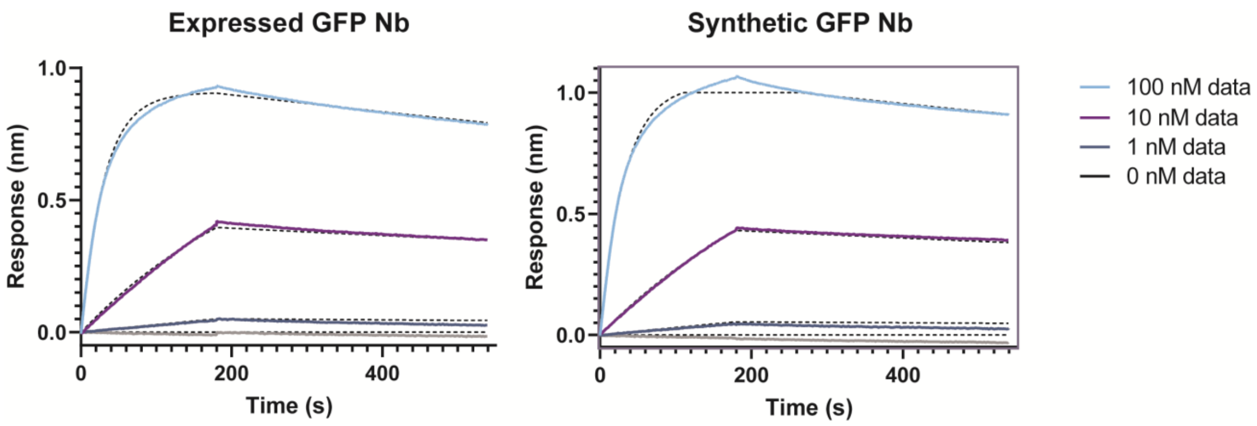

Figure S17. BLI data for binding of the expressed GFP Nb to GFP and the synthetic GFP Nb to GFP.

Pull-down

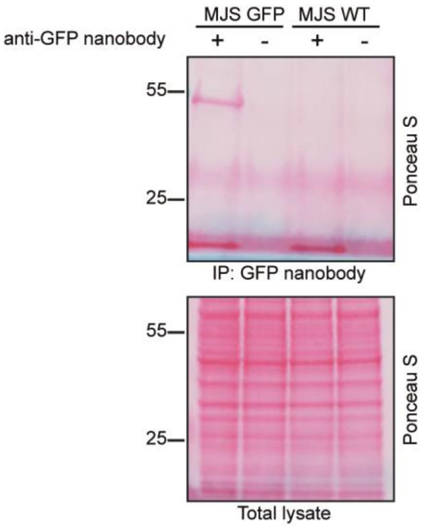

Figure S18. Ponceau S staining of the GFP-Rab7 pull-down. Signal above the 25 kDa marker is streptavidin which is released from the streptavidin beads.

References

[1] M. H. Kubala, O. Kovtun, K. Alexandrov, B. M. Collins, *Protein Sci.* **2010**, *19*, 2389–2401.

[2] A. Sapmaz, I. Berlin, E. Bos, R. H. Wijdeven, H. Janssen, R. Konietzny, J. J. Akkermans, A. E. Erson-Bensan, R. I. Koning, B. M. Kessler, J. Neefjes, H. Ovaa, *Nat. Commun.* **2019**, *10*, 1–18.

[3] N. Rocha, C. Kuijl, R. Van Der Kant, L. Janssen, D. Houben, H. Janssen, W. Zwart, J. Neefjes, *J. Cell Biol.* **2009**, *185*, 1209–1225.

[4] M. L. Jongsma, J. Bakker, B. Cabukusta, N. Liv, D. Elsland, J. Fermie, J. L. Akkermans, C. Kuijl, S. Y. Zanden, L. Janssen, D. Hoogzaad, R. Kant, R. H. Wijdeven, J. Klumperman, I. Berlin, J. Neefjes, *EMBO J.* **2020**, *39*, 1–25.

[5] B. Cabukusta, I. Berlin, D. M. van Elsland, I. Forkink, M. Spits, A. W. M. de Jong, J. J. L. L. Akkermans, R. H. M. Wijdeven, G. M. C. Janssen, P. A. van Veelen, J. Neefjes, *Cell Rep.* **2020**, *33*, 108475.

[6] C. M. Guardia, R. De Pace, A. Sen, A. Saric, M. Jarnik, D. A. Kolin, A. Kunwar, J. S. Bonifacino, *PLoS Biol.* **2019**, *17*, 1–27.

[7] A. M. Moorhead, J. Y. Jung, A. Smirnov, S. Kaufer, M. A. Scidmore, *Infect. Immun.* **2010**, *78*, 1990–2007.

[8] L. A. Carpino, E. Krause, C. D. Sferdean, M. Schümann, H. Fabian, M. Bienert, M. Beyermann, *Tetrahedron Lett.* **2004**, *45*, 7519–7523.

[9] M. Mutter, A. Chandravarkar, C. Boyat, J. Lopez, S. Dos Santos, B. Mandal, R. Mimna, K. Murat, L. Patiny, L. Saucède, G. Tuchscherer, *Angew. Chemie - Int. Ed.* **2004**, *43*, 4172–4178.

[10] T. Yoshiya, H. Kawashima, Y. Sohma, T. Kimura, Y. Kiso, *Org. Biomol. Chem.* **2009**, *7*, 2894–2904.
